# Treatment of *Schistosoma mansoni* with miltefosine *in vitro* enhances serological recognition of defined worm surface antigens

**DOI:** 10.1371/journal.pntd.0005853

**Published:** 2017-08-25

**Authors:** Marwa H. El-Faham, Maha M. Eissa, Joseph E. Igetei, Eglal I. Amer, Susan Liddell, Mervat Z. El-Azzouni, Michael J. Doenhoff

**Affiliations:** 1 Department of Medical Parasitology, Faculty of Medicine, Alexandria University, Alexandria, Egypt; 2 School of Life Sciences, University Park, University of Nottingham, Nottinghamshire, Nottingham, United Kingdom; 3 Department of Animal and Environmental Biology, Faculty of Life Sciences, University of Benin, Benin City, Edo State, Nigeria; 4 School of Biosciences, Sutton Bonington Campus, University of Nottingham, Nottinghamshire, Nottingham, United Kingdom; George Washington University, UNITED STATES

## Abstract

**Background:**

Miltefosine, an anti-cancer drug that has been successfully repositioned for treatment of *Leishmania* infections, has recently also shown promising effects against *Schistosoma* spp targeting all life cycle stages of the parasite. The current study examined the effect of treating *Schistosoma mansoni* adult worms with miltefosine on exposure of worm surface antigens *in vitro*.

**Methodology/Principal findings:**

In an indirect immunofluorescence assay, rabbit anti-*S*.*mansoni* adult worm homogenate and anti-*S*. *mansoni* infection antisera gave strong immunofluorescence of the *S*. *mansoni* adult worm surface after treatment with miltefosine, the latter antiserum having previously been shown to synergistically enhance the schistosomicidal activity of praziquantel. Rabbit antibodies that recognised surface antigens exposed on miltefosine-treated worms were recovered by elution off the worm surface in low pH buffer and were used in a western immunoblotting assay to identify antigenic targets in a homogenate extract of adult worms (SmWH). Four proteins reacting with the antibodies in immunoblots were purified and proteomic analysis (MS/MS) combined with specific immunoblotting indicated they were the *S*. *mansoni* proteins: fructose-1,6 bisphosphate aldolase (SmFBPA), Sm22.6, alkaline phosphatase and malate dehydrogenase. These antibodies were also found to bind to the surface of 3-hour schistosomula and induce immune agglutination of the parasites, suggesting they may have a role in immune protection.

**Conclusion/Significance:**

This study reveals a novel mode of action of miltefosine as an anti-schistosome agent. The immune-dependent hypothesis we investigated has previously been lent credence with praziquantel (PZQ), whereby treatment unmasks parasite surface antigens not normally exposed to the host during infection. Antigens involved in this molecular mechanism could have potential as intervention targets and antibodies against these antigens may act to increase the drug’s anti-parasite efficacy and be involved in the development of resistance to re-infection.

## Author summary

Schistosomiasis (Bilharzia) is a serious public health problem caused by a parasite of genus *Schistosoma*. There is an increasing concern about development of parasite resistance to the only drug available for treatment, praziquantel (PZQ). Miltefosine, a repurposed anti-cancer drug for treatment of *Leishmania* infection, was shown to have activity against *Schistosoma* in animal models at all the parasite’s life cycle stages. In this work, we examined the potential that miltefosine could act to expose parasite surface antigens that are normally hidden during natural infection as a way to avoid lethal effects of host immunity. We used two immunobinding techniques, immunofluorescence and western immunoblotting, and a protein identification technique, namely mass spectrometry, to identify proteins exposed on the worm surface following incubation with miltefosine. Four *S*. *mansoni* proteins were shown to be exposed by miltefosine treatment: fructose-bisphosphate aldolase (SmFBPA), Sm22.6, alkaline phosphatase and malate dehydrogenase. Antibodies specific for these antigens recognised and bound to the surface of early-stage schistosome larvae and antibodies specific for SmFBPA induced clumping of the larvae, suggesting a potential role in early parasite killing and protection against infection. These antibodies may be utilised to increase miltefosine’s anti-parasite efficacy and may be involved in resistance to re-infection.

## Introduction

Despite extensive control efforts, schistosomiasis is still prevalent in many countries [1]. Concerns are growing over development of resistance to the only drug available for treatment, namely praziquantel (PZQ), because of its extensive use in control programs for almost three decades [2,3]. The suboptimal efficacy of PZQ against immature stages of the infection is another concern [4]. Thus, the search for an alternative therapeutic control or a combination strategy that is effective against multiple stages of the schistosome life cycle has become necessary [5,6]. One approach that may accelerate discovery of new drugs is to identify and develop alternative uses for existing, approved drugs that have had their pharmacokinetics, toxicity and pre-clinical data evaluated − an approach known as ‘Drug-Repositioning’ [7,8]. One promising example is miltefosine, a hexa-decyl-phosphocholine discovered in the late 1980s and approved as Miltex in 1992 for topical treatment of cutaneous metastasis of breast cancer [9,10]. The drug later demonstrated effectiveness against antimony-resistant *Leishmania* [11,12] and (under the trade name Impavido) was approved for oral treatment of visceral leishmaniasis in some countries [13–15].

Recent studies by Eissa *et al*. [16–18], Bertao *et al*. [19,20] and El-Moslemany *et al*. [21] demonstrated significant effectiveness of miltefosine against *Schistosoma mansoni* (*S*. *mansoni*) and *Schistosoma haematobium in vitro* and against different developmental stages of *S*. *mansoni in vivo*. The drug exhibited an advantage over PZQ by clearing the infection at the invasive, immature and adult parasite stages [4,17]. The mechanism by which miltefosine exerts its effect is unclear, although it is believed to act mainly on cell membranes producing biophysical perturbation of lipid rafts and interfering with phospholipid metabolism and cell growth signalling pathways, subsequently promoting cell apoptosis [19,22–24]. One of the drug targets is sphingomyelin, a schistosome membrane phospholipid (ceramide phosphorylcholine) [25]. Biosynthesis of this molecule was shown to be impeded by miltefosine [25]. Sphingomyelin acts to conceal surface membrane proteins from the host immune system through formation of a tight barrier of hydrogen bonds with water molecules [26]. Evidence of damage to the schistosome tegument following treatment with miltefosine has been referred to in several reports [16–20], but no studies have yet been conducted to clearly establish if the drug induces exposure of antigens on the parasite surface.

In the present work, we evaluated the *in vitro* effect of miltefosine, relative to PZQ, on a Puerto Rican strain of *S*. *mansoni* and investigated whether the drug treatment would ‘unmask’ hidden antigens on the adult worm tegument using immunofluorescence (IF) and western immunoblotting assays linked to a proteomic approach i.e. tandem mass spectrometry (MS/MS). The likelihood of expression of the proteins exposed by the drug on the surface of adults and 3-hour schistosomula has also been investigated. Exposure of cryptic adult membrane proteins has previously been reported after treatment with PZQ and other anti-schistosomal drugs [27–34], an event which was thought to be an important factor in host immune responses directed at intact worms and to be potentially involved in development of resistance to re-infection by targeting migratory schistosomula [35–37].

## Methods

### Ethics statement

All work with laboratory animals, including maintenance of life cycle of *Schistosoma mansoni* by repeated passage through random-bred CD1 strain mice and laboratory-bred *Biomphalaria glabrata* snails, was conducted according to the UK Animal (Scientific Procedures) Act 1986 with personal and project license authorities held by MJD (numbers PIL 70/3255 and PPL 40/3024 respectively). Work performed under these authorities was approved by Nottingham University’s Animal Welfare and Ethical Review Body and was carried out in strict accordance with UK government regulations for animal welfare and amelioration of suffering in force at the time. The biological materials obtained from the animals under the above authorities was utilised for immunobinding experiments in accordance with the Egyptian National Animal Welfare Standards, approved by the Alexandria University’s Faculty of Medicine Ethics Committee (protocol approval number 0303186).

### Chemicals and drug

All chemicals were purchased from Sigma-Aldrich (UK) unless otherwise indicated. Miltefosine (hexa-decyl-phosphocholine) was purchased from Calbiochem (Cambridge, UK) and praziquantel (PZQ) from Merck Ltd (UK). For *in vitro* treatment, drugs were dissolved in a minimal volume of 0.5% dimethyl sulfoxide (DMSO) diluted in Roswell Park Memorial Institute (RPMI) 1640 medium (Invitrogen, UK) to generate a stock solution of 5 mg/ml [16,38,39].

### Parasites and animals

A Puerto Rican strain of *S*. *mansoni* was maintained by repeated passage through random-bred female CD1 strain mice and laboratory-bred *Biomphalaria glabrata* snails using previously described methods [40]. The life cycle of the parasite has been maintained in the laboratory for more than 40 years. The *S*. *mansoni* strain used in the present study was originally isolated from eggs obtained from a Puerto Rican patient in 1960s by Dr. Smithers SR, NIMR, London. This strain was analyzed anonymously and is currently kept at the University of Nottingham. Cercariae were used for animal infection or mechanically transformed into schistosomula as previously described [41]. Mice were percutaneously infected with 200 cercariae using methods previously described [29,42]. 42 days after infection, animals were sacrificed using an overdose of pentobarbitone anaesthetic and worms were recovered from their portal system as described elsewhere [43].

### Preparation of aqueous parasite extract

A crude protein extract from *S*. *mansoni* adult worms (whole worm homogenate), SmWH, was prepared according to methods previously described by Doenhoff *et al*. [44]. *S*. *mansoni* adult worms washed in phosphate-buffered saline (PBS) were mechanically homogenized using a ground-glass homogenizer, and the homogenate centrifuged at 2000 x g for 10 minutes at room temperature. The supernatant was collected, freeze-dried and kept at −80°C. To prepare *S*. *mansoni* detergent-soluble adult worm extract, freshly perfused worms were incubated in PBS, pH 7.4, the gravity-sedimented worm pellet being resuspended in an equal volume of buffer containing 2% sodium deoxycholate [44]. The suspension was gently agitated on a rocker for 3 hours at room temperature, and then centrifuged at 5,000 x g for 30 minutes at room temperature. The supernatant was collected, freeze-dried and kept at −80°C. When required for use, samples were reconstituted with distilled water and the protein concentration was measured by the BioRad DC Protein Assay (BioRad Laboratories, Inc., Hercules, California USA) using bovine serum albumin as the reference standard.

### Rabbit anti-*S*. *mansoni* antisera

A polyspecific rabbit anti-*S*. *mansoni* infection antiserum, anti-SmI, was developed as described by Doenhoff *et al*. [44] by exposing New Zealand white rabbits (B&K Universal, UK) to 5 rounds of percutaneous *S*. *mansoni* infections, each of approximately 15 x 10^3^ cercariae, via the ear at two week intervals. Two weeks after the last cercarial exposure, 3 samples of 20 ml blood were removed from an ear vein at two-week intervals after which the rabbits were exsanguinated by cardiac puncture. Sera from the serial and final bleeds were pooled and stored at −20°C until required. A rabbit antiserum polyspecifically reactive against *S*. *mansoni* adult worm homogenate (anti-SmWH) was prepared as described before [44,45]. New Zealand white rabbits were immunized subcutaneously at two week intervals with 1 ml of the homogenate containing approximately 5 mg protein emulsified with an equal volume of Freund’s complete (CFA) and incomplete adjuvants for the first and subsequent booster injections, respectively. The rabbits were serially bled via an ear vein every two weeks and when sera showed what was considered sufficient immunoreactivity in an immunoelectrophoretic analysis, animals were serially bled, followed by terminal anaesthesia and exsanguination by cardiac puncture. Sera from serial and final bleeds were pooled and stored at −20°C until needed. Rabbit antiserum monospecifically reactive against *S*. *mansoni* alkaline phosphatase (SmAP) was prepared by immunization of rabbits subcutaneously with replicates of immunoprecipitin arcs containing the enzyme excised from immunoelectrophoresis gels, homogenized and emulsified with an equal volume of Freund’s adjuvants [45,46]. The immunization protocol was as described above for anti-SmWH. Sera from rabbits injected with Freund’s adjuvants alone, developed as above, and normal rabbit sera from naive uninfected animals were used as controls for the IF experiments. The rabbit antibodies were prepared using Freund's Complete Adjuvant during the period from 1990- to 2000, before a ban was placed on the use of CFA in Europe [47].

### *In vitro* miltefosine and praziquantel treatment and immunofluorescence (IF) of schistosome adult worms

Intact freshly-recovered *S*. *mansoni* adult worms (males/couples) obtained by perfusion from infected mice were rinsed in RPMI-1640 medium (supplemented with 5% fetal calf serum (FCS), 2 mM L-glutamine, 100 μg/ml streptomycin and 100 IU/ml penicillin), placed in a 24-well culture plate containing 1 ml/well of the supplemented RPMI-1640 medium and incubated at 37°C in 5% CO_2_ incubator for 2 hours to recover, the method being adapted from Eissa *et al*. [16], Bertao *et al*. [19,20] and Mossallam *et al*. [48]. Worms were divided into three groups, each being tested in triplicate, as follows: Group I, untreated worms in a medium containing 0.5% DMSO (vehicle) were used as negative controls (n = 10); Group II, worms treated with PZQ; Group III, worms treated with miltefosine. Groups II and III were further subdivided into 5 and 4 subgroups, respectively (n = 10 for each) to test different concentrations of the PZQ: 2, 4, 6, 8 and 10 μg/ml [38] and the miltefosine: 5, 10, 20 and 40 μg/ml [16]. All groups were incubated at 37°C in 5% CO_2_ incubator for 96 hours. Worms were examined under an inverted microscope (Olympus Inverted Microscope Model IX70, Olympus, Tokyo, Japan) every 24 hours and observed for changes in their motility, pairing, tegument and for mortality, and the data was analysed with respect to both the drug concentration and the incubation time. Mortality was indicated by absence of any body movement detected during 2 minutes of observation [49,50]. Lethal concentrations of miltefosine that killed 50% of worms (LC50) at the examination times of 24, 48, 72 and 96 hours were calculated using Excel Microsoft software and a curve for LC50s at different time points was generated using GraphPad Prism 4 software (Inc., La Jolla—CA, USA).

After 96 hours or in the case of death, worms from all groups were rinsed three times in RPMI medium, fixed in 4% paraformaldehyde (PFA) in PBS, pH 7.4 overnight at 4°C, washed thoroughly three times in PBS, each for 5 minutes. Fixed worms were blocked by incubation with 1% bovine serum albumin (BSA) in PBS-Tween (PBST) (0.5% v/v Tween20 in PBS) for 1 hour at room temperature and washed as above in PBST. Worms from untreated Group I and all subgroups in groups II and III were further subdivided into two subgroups/(sub)group, each was tested in triplicate wells (n = 5/well), incubated with the following heat-inactivated (56°C for 30 minutes) rabbit sera: anti-*S*. *mansoni* infection antiserum (anti-SmI); anti-*S*. *mansoni* worm homogenate antiserum (anti-SmWH). All rabbit sera were diluted 1:20 in PBST and incubated with the worms overnight at 4°C. After washing in PBST, fluorescein isothiocyanate (FITC)-conjugated goat anti-rabbit IgG antibodies (diluted 1:40) were added for 30 minutes in the dark at room temperature as previously described [51]. Labelled worms were examined under ultraviolet light microscopy (GMXL3201LED) and worms displaying representative staining patterns were photographed using a GXCAM-FLUOMA-X camera (GT Vision Ltd) and GXCapture7 software. All images were obtained under consistent microscope settings.

### Elution of rabbit antibodies bound to the schistosome adult worm surface

120 worms freshly perfused from *S*. *mansoni*-infected mice at day 42 after infection were divided into three groups (n = 40/group): Group I, untreated; Groups II and III, treated with the drug dose and for the duration that produced the greatest changes in worm tegument *in vitro* and the most intense immunofluorescence (6 μg/ml PZQ for 24 hours and 40 μg/ml miltefosine for 48 hours). Treatment of worms, washings, fixation in 4% PFA and blocking were carried out as described above. Worms in each group were then divided into four subgroups (n = 10 for each), (A): incubated with the rabbit anti-SmI serum; (B): incubated with the rabbit anti-SmWH serum; (C): incubated with a control serum from rabbits that received adjuvant alone; (D): incubated with a control serum from naive rabbits. Before use, rabbit sera were heat inactivated for 30 minutes at 56°C, diluted 1:20 in PBST and incubated with the worms overnight at 4°C. Four worms from each subgroup were incubated with 1:40 FITC-conjugated goat anti-rabbit IgG and examined under the fluorescence microscope to check for antibody binding. The rest of the worms were used to recover rabbit antibodies bound to their surfaces from the groups that showed immunofluorescence staining using a method adapted from Doenhoff *et al*. [52] for elution of antibodies from antigens fixed to a nitrocellulose film. After incubation with the primary rabbit sera worms were washed thoroughly three times in PBST, pH 7.4, each for 5 minutes. The worms were then incubated in a low-pH buffer, 0.1 M glycine, pH 2.8 for 10 minutes to elute off surface-bound antibodies, after which the eluates were collected and neutralized using 0.1 ml of 1M Tris-HCl, pH 8.0. The process of incubation with primary antibody, washing and antibody-elution using low pH buffer was repeated up to 4 times. The eluates from each subgroup were pooled, concentrated to 5% of the starting volume using Amicon ultra centrifugal filters, 3000 molecular weight cut-off (Millipore, Corrigtwohill, Co. Cork, Ireland), aliquoted and kept frozen at -20°C until needed.

### Western immunoblotting for detection of protein targets in *S*. *mansoni* worm extract

Protein samples (10 μg/lane) from *S*. *mansoni* adult worm crude extract were analyzed in reducing 10% or 8% SDS-PAGE gels prepared as described elsewhere [53,54]. SDS-PAGE gels were run using a Bio-Rad Mini Protean II electrophoresis system (Bio-Rad Laboratories, California, USA) and were stained with SimplyBlue SafeStain (Invitrogen, UK) according to the manufacturer’s instructions. Proteins resolved with SDS-PAGE were transferred to nitrocellulose paper (NCP) strips at 50 V for two hours as previously described [45,55]. After visualizing the protein marker, the membranes were blocked with 5% skim milk in TBST (TBS-0.5% v/v Tween20) overnight at 4°C. Blot strips were washed x3 in TBST and probed with the primary rabbit antibodies (diluted 1:100 in TBST) for two hours at room temperature. After washing, the membranes were incubated with the secondary antibody: horse radish peroxidise (HRP)-conjugated goat anti-rabbit IgG antibody (Sigma, UK) diluted 1:1000 for 2 hours at room temperature. The immunoblots were then developed using 4-chloro-1-naphthol substrate (Sigma, UK) as described by the manufacturer.

### Immunofluorescence for detection of reactivity of antibodies recovered from drug-treated adult worms on the surface of schistosomula

Schistosomula that had been mechanically transformed from freshly-harvested cercariae [41] were incubated in RPMI-1640 medium supplemented with 5% FCS, 2 mM L-glutamine, 100 μg/ml streptomycin and 100 IU/ml penicillin in a 24-well culture plate containing about 500 parasites in 1ml/well medium at 37°C, 5% CO_2_ for 3 hours, then fixed in 4% PFA in PBS for 1 hour at room temperature. Thereafter, the parasites were transferred to 5 ml glass test tubes, blocked by incubation in 1% BSA in PBST for 1 hour at room temperature. Fixed, blocked schistosomula were incubated with rabbit anti-SmI or anti-SmWH antibodies eluted from PZQ or miltefosine-treated worms. Control groups included schistosomula incubated with heat-inactivated sera from naive rabbits and from rabbits that had received adjuvant alone. Parasites were incubated with the eluted rabbit antibodies or control sera diluted 1:20 overnight at 4°C, washed three times in PBST and incubated with FITC-conjugated goat anti-rabbit IgG antibodies diluted 1:40 in PBST for 30 minutes in the dark at room temperature. IF staining was evaluated by fluorescence microscopy as described above. At least 40 parasites were observed for each group and those displaying representative staining patterns were photographed.

### Antigen purification

About 150–200 μg of SmWH was placed in broad wells (6.2 cm long) and fractionated in reducing 12% SDS-PAGE gels and the gels were stained with the SimplyBlue SafeStain. Target proteins were identified by matching western blots bearing the immunoreactivities of interest to protein bands in SimplyBlue-stained SDS-PAGE gels. The proteins were purified by excision of the respective bands and elution of the proteins from the gel by incubation in a buffer containing 10% SDS as previously described [56,57]. The eluates were centrifuged at 14,000 x g, 37°C for 30 minutes and kept frozen at −20°C until use. Solutions of eluted purified proteins were concentrated using Amicon ultra centrifugal filters, 3000 molecular weight cut-off (Millipore, Corrigtwohill, Co. Cork, Ireland), their protein concentrations measured using the BioRad DC Protein Assay (BioRad Laboratories, USA) and their molecular weights and antigenic reactivity were confirmed by SDS-PAGE and western blotting. Purified, concentrated proteins were stored at −20°C.

### Tandem mass spectrometry (MS/MS) analysis of purified proteins

SDS-PAGE gel slices bearing the target proteins were manually and individually excised, de-stained, reduced (DTT), carbamidomethylated (iodoacetamide) and digested with trypsin (20 μg/ml overnight at 37°C). The resulting peptides were submitted to tandem mass spectrometry (MS/MS) for protein identification [58]. The MS/MS data were used to search the public database NCBInr (version 20140927) using the Mascot software, version 2.3.01 (Matrixscience, UK, http://www.matrixscience.com) for peptide matching [59].

## Results

### Miltefosine pre-treatment of Puerto Rican *S*. *mansoni* adult worms induces tegumental changes and exposure of surface proteins

**Fig 1:**
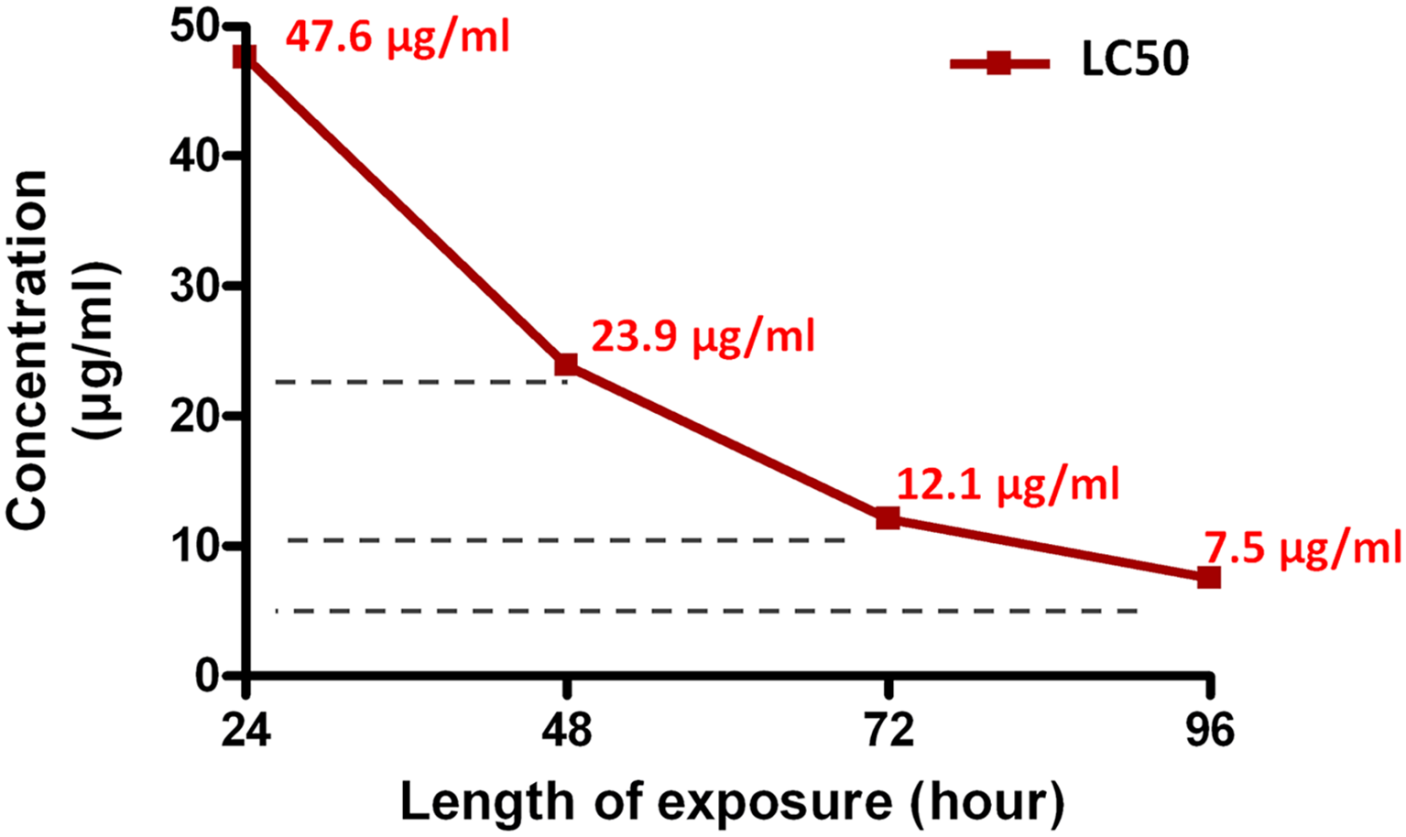
*In vitro* lethal effect of miltefosine on *S*. *mansoni* adult worms. LC50s of the drug (concentrations that kill 50% of worms) 24, 48, 72 and 96 hours after *in vitro* exposure to miltefosine were calculated from concentration-response curves at the respective time points and LC50 values were plotted against length of incubation.

The effect of *in vitro* treatment of *S*. *mansoni* with miltefosine was evaluated every 24 hours over a time-course of 96 hours at different drug concentrations (5–40 μg/ml) relative to the control untreated and PZQ-treated worms. All control worms (incubated in supplemented RPMI medium containing 0.5% DMSO) survived and exhibited normal appearance with no tegumental changes throughout the examination period (Table 1) consistent with previously described results [50,60]. Parasites in the control group showed natural peristaltic motility of the body and were occasionally seen attached to the bottom of the well by their ventral suckers, male and female couples remaining paired. PZQ treatment at tested doses of 4–10 μg/ml resulted in instant muscular contraction with complete loss of motor activity in all worms and 90%-100% parasite mortality within 24 hours (Table 1). Light microscopic examination demonstrated tegumental disruption (S1 Fig) most obviously seen in the subgroup treated with 6 μg/ml PZQ. Extensive tegumental damage was seen with miltefosine doses of 10–40 μg/ml in 70–80% of the worms within 48–96 hours under an inverted microscope. The drug eventually resulted in worm death in 70–100% of worms (Table 1). These changes, however, developed at a slower rate than those observed with PZQ; the intensity of the effects by miltefosine was both dose- and time-dependent. The LC50 (drug concentration needed to cause 50% worm killing) in 24 hours was 47.6 μg/ml, while 23.9 μg/ml, 12.1 μg/ml and 7.5 μg/ml miltefosine were needed to kill 50% of worms in 48, 72 and 96 hours, respectively (Fig 1). Worm separation into individual males and females was observed within 24 hours in 70% of worms with miltefosine doses as low as 10 μg/ml. In contrast, only 10% of paired worms in the PZQ-treated group showed separation of couples (Table 1).

**Table 1:** *In vitro* activity of miltefosine against *S*. *mansoni* adult worms.

Group	Dose μg/ml (μM)	Hours of incubation	Number	UncoupledWorms	Dead	Reduced activity ^a^		Tegumental changes ^b^	
						+	++	+	++
**Control**		96	10	0	0	0	0	0	0
**Praziquantel**	2 (06.4)	24	10	0	05	0	5	0	0
	4 (12.8)	24	10	0	09	0	1	8	0
	6 (19.2)	24	10	1	10	0	0	3	7
	8 (25.6)	24	10	1	10	0	0	5	5
	10 (32.0)	24	10	1	10	0	0	6	4
**Miltefosine**	5 (12.5)	24	10	3	0	0	0	0	0
		48	10	7	0	0	0	0	0
		72	10	10	0	3	2	2	1
		96	10	10	0	5	5	5	2
	10 (25.0)	24	10	7	0	1	0	0	0
		48	10	8	2	1	1	2	2
		72	10	10	4	3	1	2	5
		96	10	10	7	3	0	3	7
	20 (50.0)	24	10	10	2	1	2	1	2
		48	10	10	4	2	6	3	5
		72	10	10	10	0	0	3	7
	40 (100.0)	24	10	10	4	3	2	6	4
		48	10	10	10	0	0	2	8

^a^ Dead worms were excluded. +, Slight. ++, Significant.

^b^ S1 Fig.

**Fig 2:**
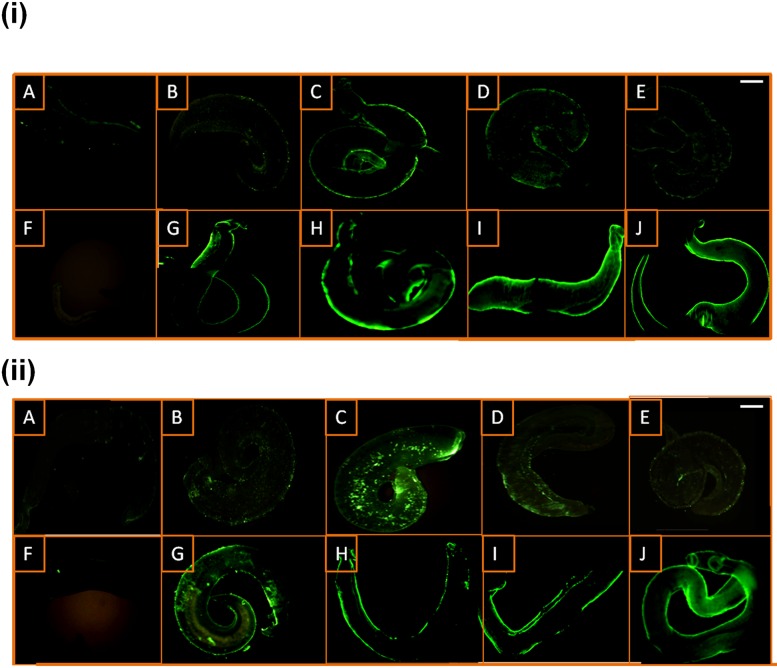
Immunofluorescent staining of miltefosine- and PZQ-treated *S*. *mansoni* adult worms. The surface of worms treated with praziquantel (PZQ) (A-E), untreated worms (F) and worms treated with miltefosine (G-J) and exposed to: rabbit anti-*S*. *mansoni* infection (anti-SmI) IgG (i), rabbit anti-*S*. *mansoni* adult worm homogenate (anti-SmWH) antisera (ii). A, 2 μg/ml PZQ; B, 4 μg/ml PZQ; C, 6 μg/ml PZQ; D, 8 μg/ml PZQ; E, 10 μg/ml PZQ; G, 5 μg/ml miltefosine; H, 10 μg/ml miltefosine; I, 20 μg/ml miltefosine; J, 40 μg/ml miltefosine. Scale bar = 100 μm.

**Fig 3:**
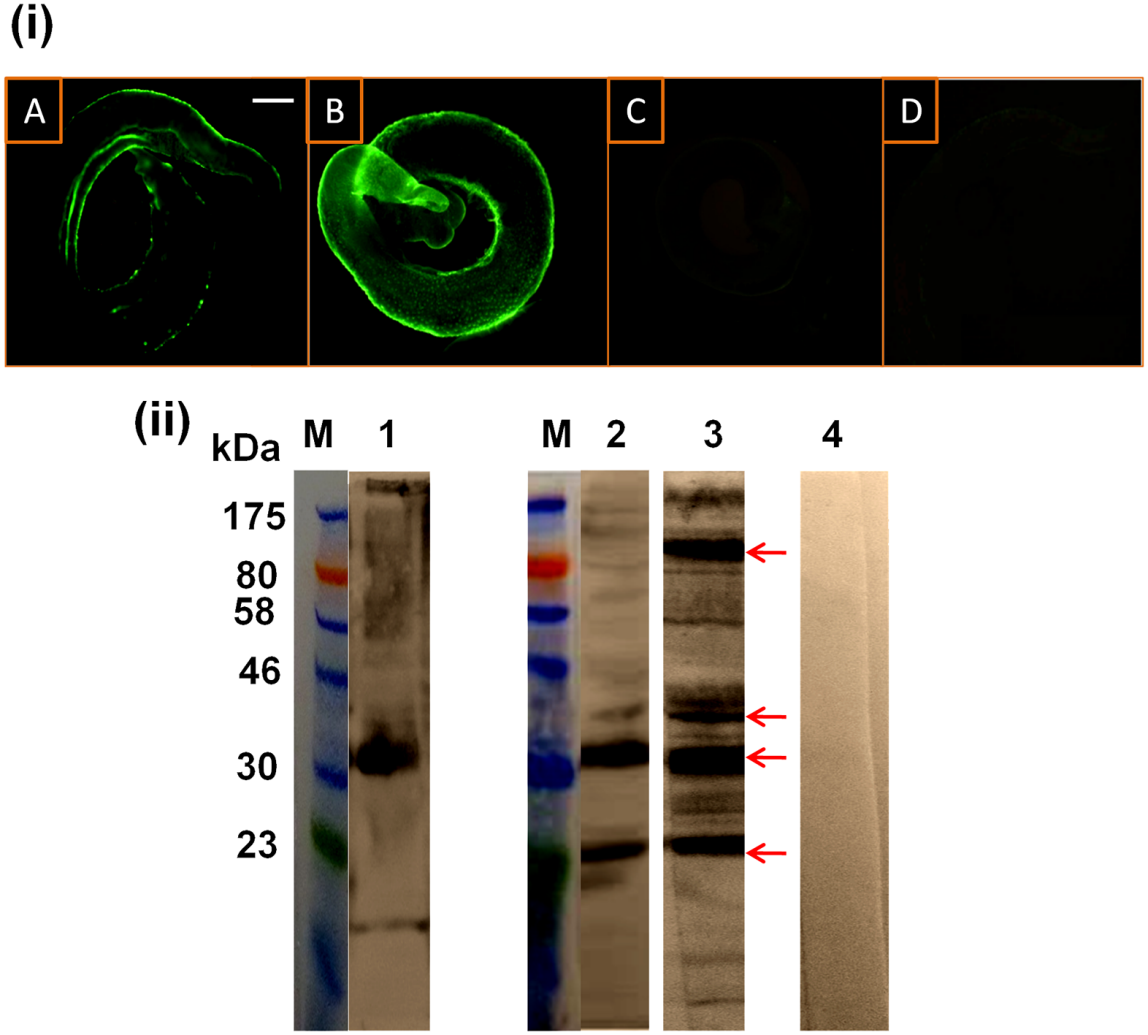
Characterization of worm surface antigens recognized by rabbit anti-SmI antibodies after miltefosine- and PZQ-treatment. (i) Immunofluorescent staining of PZQ- (6 μg/ml for 24 hours) (A and C) and miltefosine- (40 μg/ml for 48 hours) (B and D) treated *S*. *mansoni* adult worms detected with rabbit anti-SmI IgG antibodies (A and B) and IgG antibodies from a normal rabbit serum (C and D). Scale bar = 100 μm. (ii) Western immunoblots of a *S*. *mansoni* crude worm homogenate preparation (SmWH) were probed with rabbit anti-SmI antiserum (lane 1), rabbit anti-SmI antibodies eluted from PZQ- (6 μg/ml for 24 hours) (lane 2) and miltefosine- (40 μg/ml for 48 hours) (lane 3) treated worms. A blot of SmWH probed with a normal rabbit serum (lane 4) was used as a control. Lane M, protein molecular weight markers. Antigenic extract was loaded onto the gel with 10 μg protein/lane. Blots were detected using HRP-conjugated anti-rabbit IgG secondary antibodies. Detected antigens with the most intense reactivities are arrowed.

The surface of drug-treated worms was recognised by IgG antibodies from the anti-infection rabbit serum, anti-SmI (Fig 2iA–2iE & 2iG–2iI), while no staining could be seen on the untreated worms using the same antibodies (Fig 2iF). With PZQ, the fluorescence was minimal at both extremes of the dose-spectrum at 2, 4 and 10 μg/ml (Fig 2iA, 2iB and 2iE) and most intensive at 6 μg/ml (Fig 2iC). Fig 2 illustrates that, in general, the staining resulting from miltefosine treatment at all the tested doses was of a higher intensity compared to that resulting from PZQ treatment. The fluorescence was observed to be dose-dependent, most evident at 20 and 40 μg/ml miltefosine (Fig 2iI and 2iJ). Comparable results were obtained using a rabbit anti-*S*. *mansoni* worm homogenate although at relatively lower staining intensities, particularly with the PZQ treated-subgroup (Fig 2iiA–2iiE), while no staining could be detected on untreated worms (Fig 2iiF). In a second experiment, no staining was detected on PZQ-treated (6 μg/ml for 24 hours) or miltefosine-treated (40 μg/ml for 48 hours) worms by sera from a naive rabbit and a rabbit injected with Freund’s adjuvants alone (Figs 3iC and 3iD and 4iC and 4iD). The miltefosine and PZQ doses of 40 μg/ml and 6 μg/ml were, respectively found to have the most intense effects in 24 and 48 hours (Table 1 and Fig 2); these groups were therefore selected for subsequent antibody-elution experiments.

**Fig 4:**
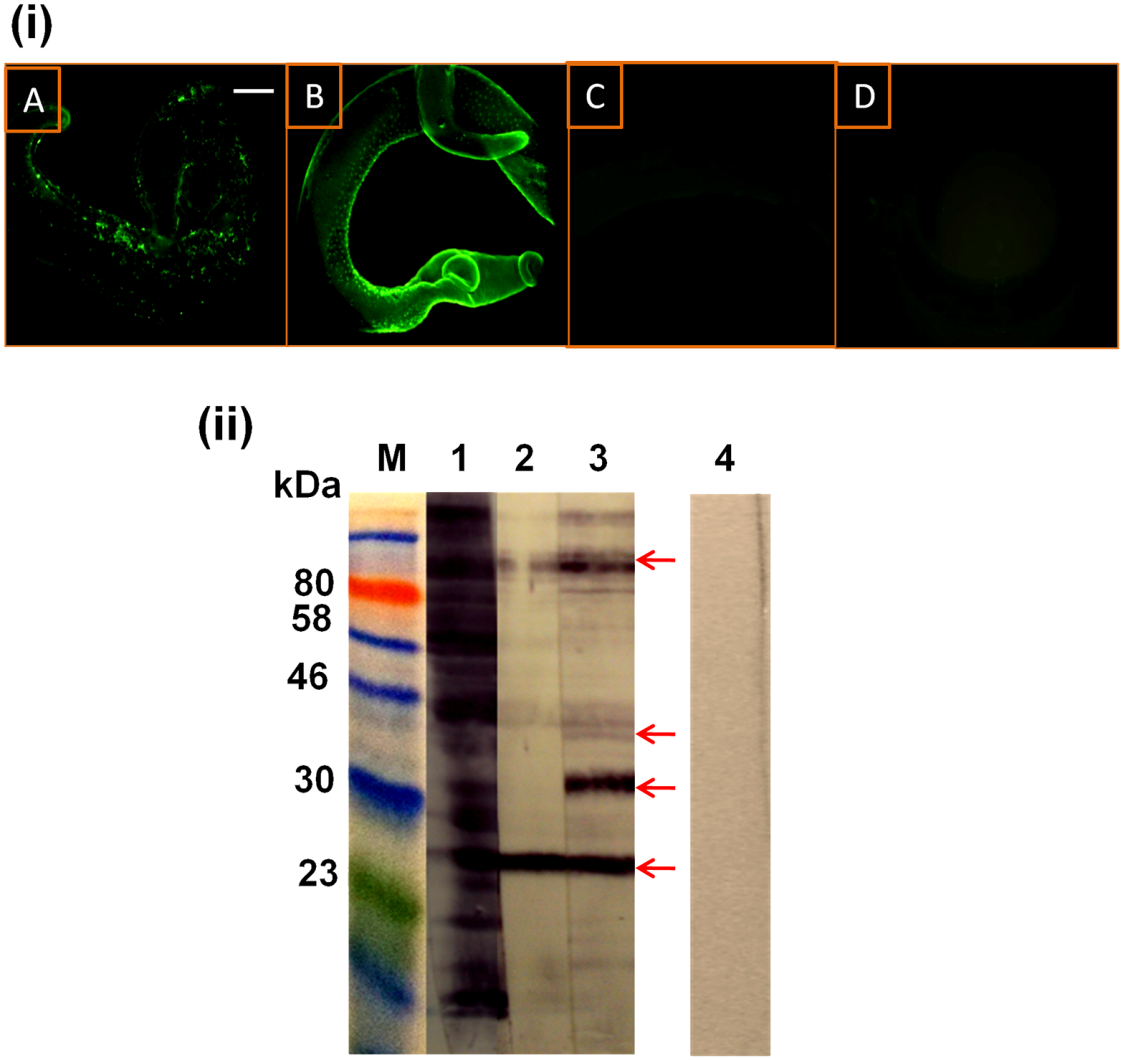
Detection by rabbit anti-SmWH antibodies of worm surface antigens exposed by miltefosine or PZQ. (i) Immunofluorescent staining of PZQ- (6 μg/ml for 24 hours) (A and C) and miltefosine- (40 μg/ml for 48 hours) (B and D) treated *S*. *mansoni* adult worms detected with rabbit anti-SmWH IgG antibodies (A and B) and IgG antibodies from a rabbit that received adjuvant alone (C and D). Scale bar = 100 μm. (ii) Western immunoblots of *S*. *mansoni* crude worm homogenate preparation (SmWH) were probed with rabbit anti-SmWH antiserum (lane 1), rabbit anti-SmWH antibodies eluted from PZQ- (6 μg/ml for 24 hours) (lane 2) and miltefosine- (40 μg/ml for 48 hours) (lane 3) treated worms. A blot of SmWH probed with a serum from a rabbit injected with Freund’s adjuvant alone (lane 4) was used as a control. Lane M, protein molecular weight markers. Antigenic extract was loaded onto the gel with 10 μg protein/lane. Blots were detected using HRP-conjugated anti-rabbit IgG secondary antibodies. Detected antigens with the most intense reactivities are arrowed.

### Proteins exposed by miltefosine and praziquantel on *S*. *mansoni* worm surface were characterized by western immunoblotting

Rabbit antibodies in the anti-SmI and the anti-SmWH sera were found to bind to the surface of worms treated earlier with 6 μg/ml PZQ for 24 hours or 40 μg/ml miltefosine for 48 hours (Figs 3iA and 3iB and 4iA and 4iB, respectively), thus confirming that treatment induced antigen exposure. No staining was detected on drug-treated worms incubated with control sera from naive and adjuvant-immunized rabbits (Figs 3iC and 3iD and 4iC and 4iD).

Surface-bound antibodies were recovered from the worm surface and used to probe western immunoblots of a crude worm extract (Figs 3 and 4, S2 and S3 Figs). The reactivity of the precursor anti-SmI antiserum with SmWH was predominantly against a ~ 33 kDa protein band (Fig 3, lane 1). Following PZQ treatment, two of these proteins (at ~23, 33 kDa) were also strongly reacted against by the anti-SmI antibodies, while antibody reactivities against the bands at ~37 and ~120–130 were of much lower intensities (Fig 3, lane 2). Our results showed that miltefosine treatment resulted in exposure of four main proteins having molecular sizes of approximately 23, 33, 37 and 120–130 kDa (the last was determined in a western immunblot transferred from an 8% gel [S4 Fig]). These protein bands were detected in blots of *S*. *mansoni* worm homogenate extract reacted against, respectively, by both the anti-SmI and the anti-SmWH antibodies that had been acid-eluted off the surface of treated worm (Figs 3 and 4, lane 3). As regards the anti-SmWH antibodies eluted after treated worms, the above four protein bands were detected following miltefosine treatment (Fig 4, lane 3), whereas in the case of PZQ, the ~33 kDa reactive band was not identified (Fig 4, lane 2). No antigenic reactivities were detected in the blots of the worm extract probed with sera from control normal/adjuvant-alone injected rabbits (Figs 3 and 4, lane 4).

**Fig 5:**
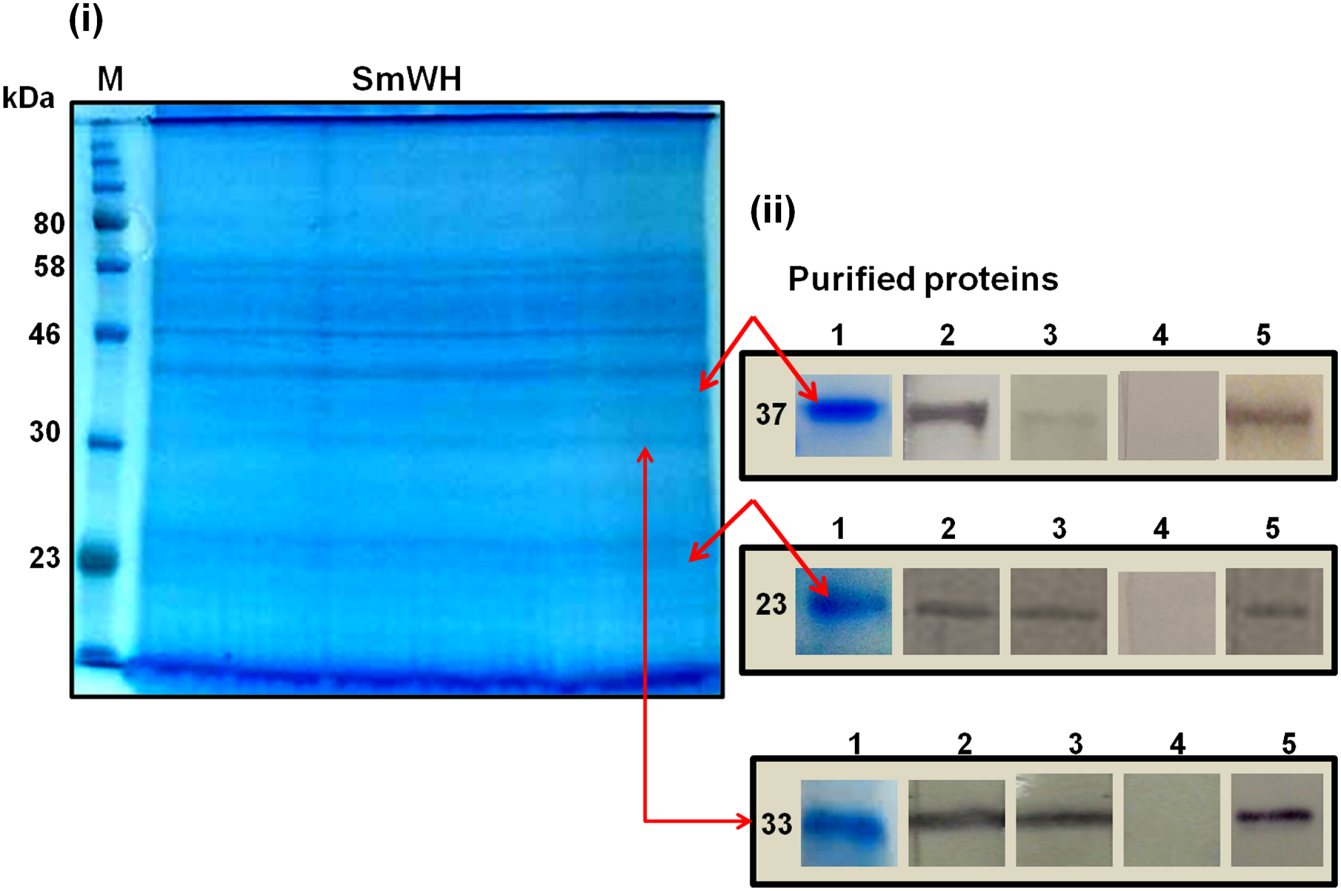
Purification and characterisation of the SmWH 23 kDa, 33 kDa and 37 kDa protein bands. (i) Coomassie blue-stained reducing 12% SDS-PAGE gel of SmWH. Protein bands at ~23, 33 and 37 kDa (arrowed) were purified from by excision and elution from the gel. Lane M, protein molecular weight marker. (ii) Purified 37, 33 and 23 kDa protein bands in Coomassie blue-stained SDS-PAGE gel (1) and western immunoblots (2–5) probed with anti-SmI eluted antibodies from miltefosine- (2) and PZQ- (3) treated worms, a normal rabbit serum (4) and a rabbit anti-SmI antiserum (5). Samples were loaded onto the gel with 10 μg protein/lane.

The protein bands at ~23 (pr23) and ~37 (pr37) kDa in the SmWH extract were purified by excision and elution from the gel films used for SDS-PAGE (Fig 5). The immunodominant band detected following treatment with miltefosine at ~33 kDa (pr37), and similarly recognised by anti-SmI on PZQ-treated parasites, was also purified from SDS-PAGE bearing the fractionated crude worm proteins. After these purification steps the three protein bands pr23, pr33 and pr37 were antigenically reactive in western blots with solutions of respective anti-SmI and anti-SmWH antibodies eluted from treated worms (Fig 5ii and S5–S7 Figs).

### MS/MS analysis of the detected protein bands pr33, pr23 and pr37

Purified protein bands at ~33 kDa, ~23 and 37 kDa from SmWH were subjected to MS/MS mass spectrometry. Mascot identification output of fragmented peptides in the reactive bands was searched against NCBInr database with the tandem MS data and found to best match, respectively *S*. *mansoni* fructose-bisphosphate aldolase (SmFBPA), *S*. *mansoni* tegument protein Sm22.6 and *S*. *mansoni* malate dehydrogenase (SmMLD) having significant Mascot scores of 1085 (S1 Table), 1262 (S2 Table) and 469 (S3 Table), respectively. The SmFBPA has a calculated mass of 39963 da (Uniprot Smp_042160, gi: 1703248. The Sm22.6 (pr23) has a calculated mass of 22563 da (UniProtKB P14202, gi: 135578,) and SmMLD (pr37) has a mass of 36305 da (UniProtKB G4V7Z7, gi: 353230847), thus matching the molecular masses of the reactivities detected in western immunoblots (Figs 3 and 4).

### Identification of the protein band at ~120–130 kDa as *S*. *mansoni* alkaline phosphatase

**Fig 6:**
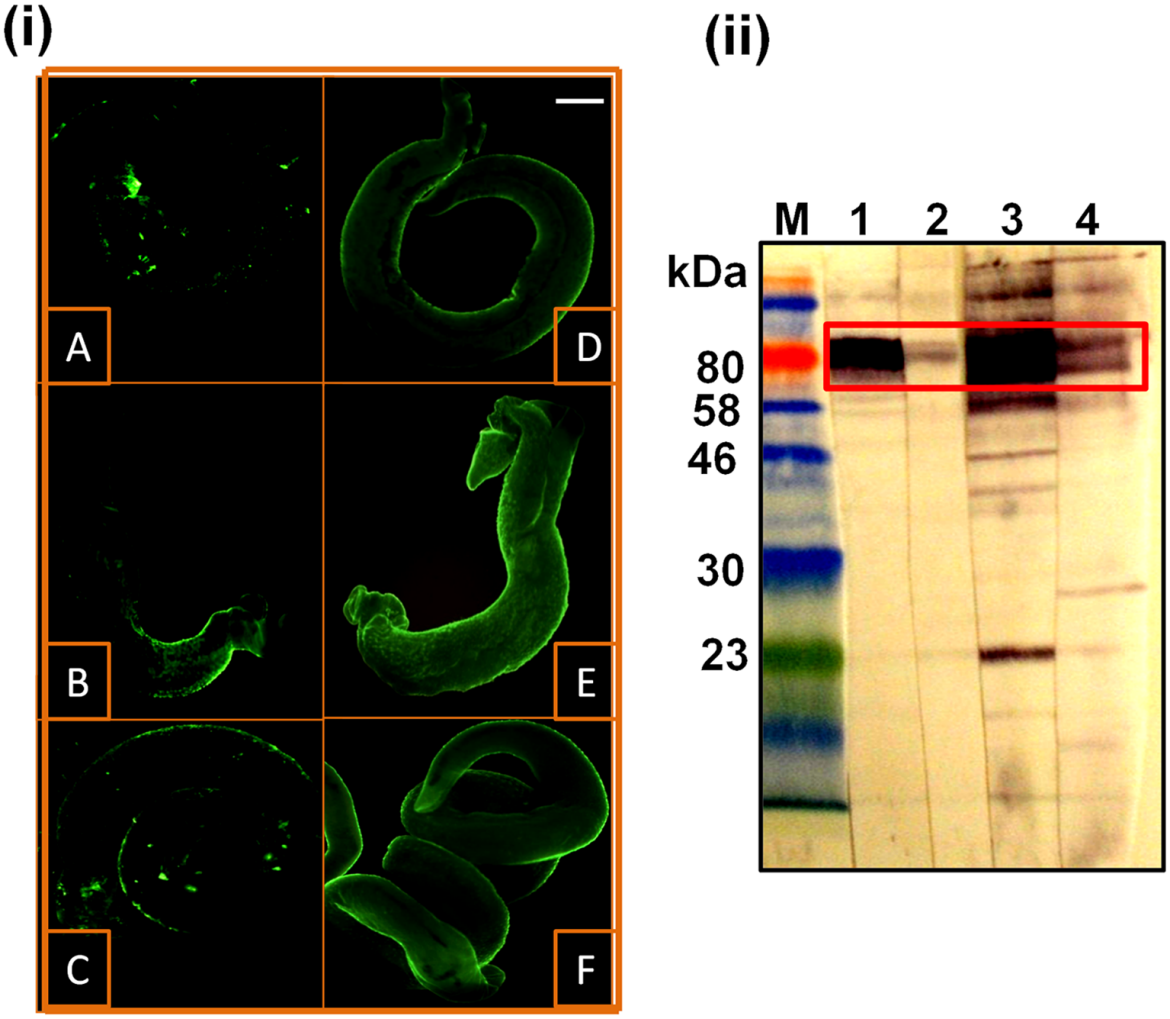
Detection of *S*. *mansoni* alkaline phosphatase (SmAP) after miltefosine treatment by immunofluorescence (i) and western blotting (ii). (i) Immunofluorescence staining of drug-treated *S*. *mansoni* adult worms that had been incubated with a rabbit anti-*S*. *mansoni* alkaline phosphatase (anti-SmAP) antiserum. A, untreated worms; B, 6 μg/ml PZQ; C, 8 μg/ml PZQ; D, 10 μg/ml miltefosine; E, 20 μg/ml miltefosine; F, 40 μg/ml miltefosine. Scale bar = 100 μm. (ii) Western immunoblots of a detergent-soluble extract of *S*. *mansoni* adult worms (lanes 1 and 2) and SmWH (lanes 3 and 4) probed with the rabbit anti-SmAP-antiserum (lanes 1 and 3) and anti-SmAP antibody eluted from miltefosine-treated worms (lanes 2 and 4); M, protein molecular weight markers. Antigenic extract was loaded onto the gel with 10 μg protein/lane. Detected SmAP is boxed.

The possibility that the rabbit anti-*S*. *mansoni* antibodies eluted from treated worms could be recognising the *S*. *mansoni* alkaline phosphatase (SmAP) protein sequences in the ~120–130 kDa doublet band (a molecular size determined using 8% SDS-PAGE (S4 Fig), having a molecular mass similar to that described in the literature for a SmAP dimer of ~130 kDa [61]), was investigated. Worms treated with 40 μg/ml miltefosine for 48 hours or 6 μg/ml PZQ for 24 hours were incubated with a rabbit antiserum specific to SmAP followed by FITC-conjugated anti-IgG antibodies and examined under a fluorescence microscope. Both PZQ and miltefosine-treated worms showed IF staining of their surface, while only minimal fluorescence was detected on untreated parasites (Fig 6i). The intensity of staining was higher after miltefosine treatment than after PZQ. Anti-SmAP antibodies eluted from the surface of miltefosine-treated worms (after they were incubated with the rabbit anti-SmAP antiserum) were used to probe immunoblots of *S*. *mansoni* worm protein extracts. Reactivities at approximately 120–130 kDa were comparable, in terms of molecular mass and pattern, to those observed using the precursor antiserum (Fig 6ii) and with our earlier blots probed with anti-SmI and anti-SmWH elutions from treated worms (Figs 3 and 4). These results indicate that the protein exposed by miltefosine treatment at ~120–130 kDa is the SmAP.

### Antibodies recovered from miltefosine-treated worms induced agglutination of schistosomula and detected the parasite surface by immunofluorescence

**Fig 7:**
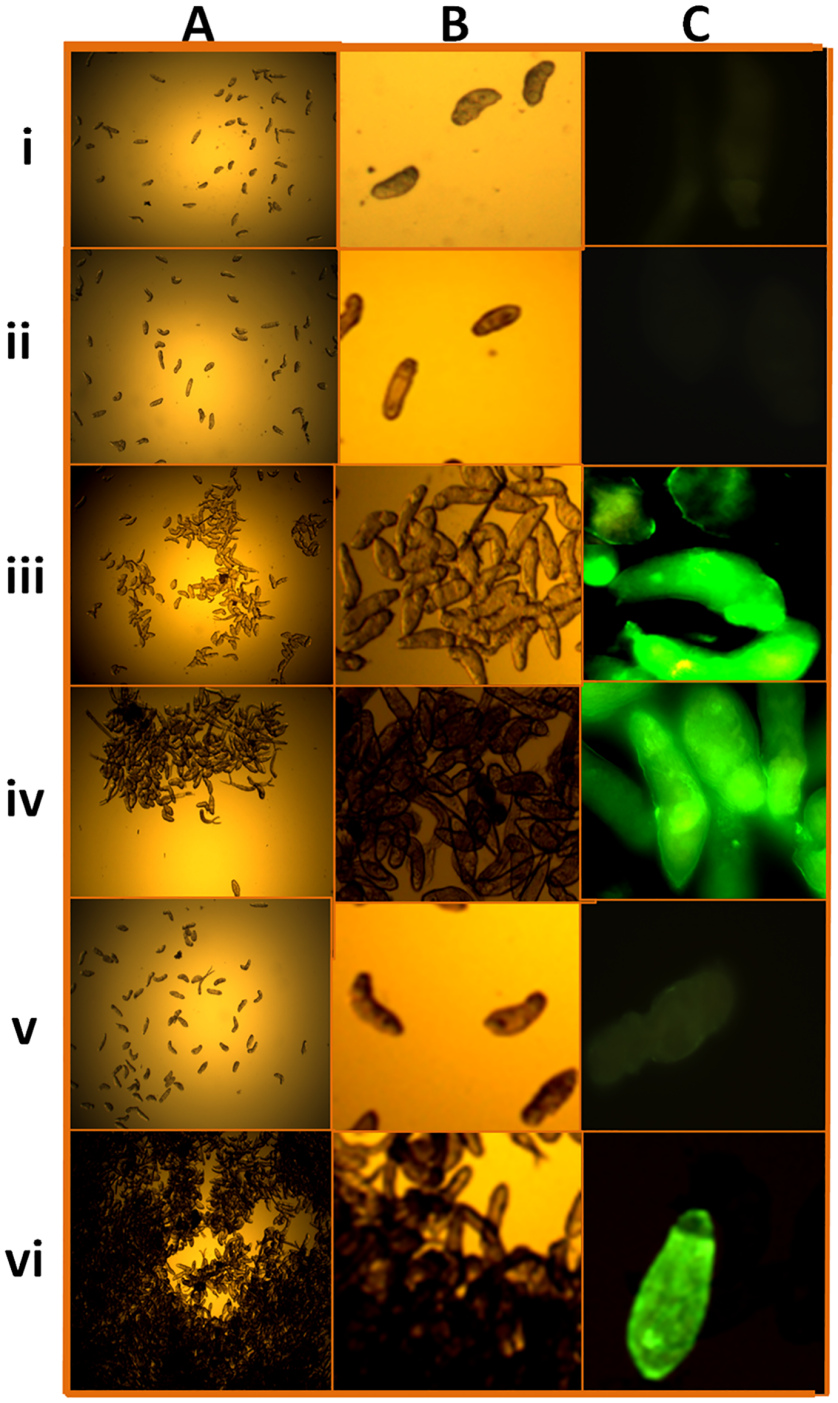
Agglutination and indirect immunofluorescence reactivity of 3-hour schistosomula with rabbit anti-*S*. *mansoni* antibodies specific for drug-exposed antigens. Representative microscopic images after incubation with the primary antibody under white light (A and B) and after processing for indirect immunofluorescence following incubation with anti-rabbit IgG conjugated to FITC (secondary antibody) (C). Schistosomula probed with: serum from naive rabbits (i); serum from rabbits immunized with adjuvant-alone (ii); anti-SmI antibodies eluted from PZQ-treated worms (iii); anti-SmI antibodies eluted from miltefosine-treated worms (iv); anti-SmWH antibodies eluted from PZQ-treated worms (v); anti-SmWH antibodies eluted from miltefosine-treated worms (vi). Scale bars = 100 μm.

An indirect immunofluorescence assay was used to examine the antigenicity of the surfaces of intact 3-hour schistosomula. Parasites were incubated with rabbit anti-SmI and anti-SmWH antibodies recovered from miltefosine- and PZQ-treated adult worms, followed by FITC-conjugated anti-IgG Fc-specific secondary antibodies. The rabbit anti-SmI and anti-SmWH antibodies eluted from miltefosine-treated worms were observed to agglutinate schistosomula (without secondary antibody), as did anti-SmI antibodies recovered from PZQ-treated worms (Fig 7). This was most obviously seen with anti-SmWH antibodies from the miltefosine-treated worms (Fig 7vi). Anti-SmWH antibodies eluted from PZQ-treated worms, and sera from naive or adjuvant-alone immunized rabbits did not have this property (Fig 7i, 7ii and 7v). Schistosomula that had agglutinated were also positive in indirect immunofluorescence labelling of their surfaces (Fig 7C).

## Discussion

Miltefosine, a hexa-phospho-choline, was previously shown to have anti-schistosomal properties and to induce extensive changes in the tegument of adult worms of Egyptian and Brazilian strains of *S*. *mansoni in vitro* and *in vivo* [16,17,19,20]. In the current study, we assessed the *in vitro* action of miltefosine on a Puerto Rican isolate of *S*. *mansoni* relative to PZQ. We particularly investigated the effect of the drug on adult worm teguments in terms of surface antigen exposure.

The present data showed that miltefosine is effective *in vitro* against adult worms of a Puerto Rican isolate of *S*. *mansoni* at concentrations of 10–40 μg/ml within 24–96 hours. These results are consistent with previous reports showing *in vitro* efficacies of miltefosine against other isolates of *S*. *mansoni* [16,19,20]. Our calculated LC50 value were 12.1 and 7.5 μg/ml miltefosine after, respectively 72 and 96 hours of incubation with drug, showing greater efficacy than those in previous studies by Eissa *et al*. [16] and Bertao *et al*. [19], who respectively reported LC50s of 5.8 μg/ml and 16 ± 0.2 μg/ml after 120 hours incubation of the Egyptian Sambon and the Brazilian BH strains. Miltefosine was conversely found to have lower effects on the *S*. *mansoni* strain ‘LE’ in a study by Yepes *et al*. [62] at the same doses we tested, though interestingly, the authors demonstrated a higher susceptibility to PZQ than that reported here. Given the reported difference in sensitivities of different strains of *Leishmania* and *Schistosoma* to miltefosine and PZQ, respectively [38,63,64], our results on the Puerto Rican strain of *S*. *mansoni* showing relatively different susceptibility to miltefosine than the Sambon, the BH and the LE strains are as expected. The possible differences in the parasite membrane properties, phospholipid composition or the drug(s) sensitive sites between different strains could be a plausible explanation [65,66]. Being effective against three schistosome strains (the Puerto Rican, the Egyptian Sambon and the Brazilian BH strains), this work further validates possible use of miltefosine as an anti-schistosomal against different *S*. *mansoni* strains. The knowledge of variation in miltefosine susceptibility of *S*. *mansoni* strains may help define drug schedules (dose/duration regimens) in future clinical trials with the drug and suggests that a laboratory pre-evaluation step is needed. Although the highest effective LC50 of the three studies that investigated miltefosine efficacy against different strains (16 μg/ml) may be the concentration to be aimed for, re-assessment of miltefosine efficacy in multiple clinical trials in various geographical areas of endemicity is needed. According to the results of the present study, it is expected that lower doses/shorter durations of treatment with miltefosine than previously reported [16,19] may be needed to treat patients with *S*. *mansoni* infection. We demonstrated that the schistosomicidal effects of miltefosine progressed slowly over 96 hours of incubation of the worms and were intensified using higher concentrations of the drug, consistent with earlier findings [16,19]. The efficacy of alkyl-phospholipids was reported earlier to depend on the drug’s contact time with the cell membrane, which could last for days [67]. PZQ, on the other hand, induced immediate muscular contraction and extensive tegumental disruption and 90%-100% parasite mortality at 6 μg/ml within 24 hours. The observed difference in time progression of drug-induced events can be accounted for by the disparate difference in the modes of action between the two drugs. On one hand, PZQ exerts its effect by inducing a rapid influx of calcium, an increase in membrane fluidity and interfering with uptake of glucose and ATP [68] resulting in worm death as soon as 3 hours [38], whereas miltefosine acts more slowly by weakly modifying lipid ordering in lipid rafts, interfering with membrane complex renewal and cell growth and survival pathways [19,22–24,69]. At doses of PZQ lower than 6 μg/ml (i.e., 2 and 4 μg/ml), no or minimal changes in worm teguments could be observed under the inverted microscope and only a little staining by IF; and at higher PZQ doses (8 and 10 μg/ml), the effects on the tegument were also seen to decrease. In accordance, Mendonca *et al*. [38] examined a range of PZQ doses *in vitro* and showed that the most extensive changes on the worm tegument occurred at 6.5 μg/ml PZQ. These findings can possibly be attributed to too low drug concentrations at 2 and 4 μg/ml (that were not enough to induce tegumental disruption) or a short time of contact with the worm tegument due to early induction of worm death at the 8–10 μg/ml levels. This early killing without significantly damaging the tegument could have resulted from the effect of PZQ on energy metabolic pathways essential to parasite survival [70] through interference with uptake of glucose and ATP or due to accumulation of lactate as a result of sustained muscle contraction [68].

In the current study, we used a rabbit anti-*S*. *mansoni* infection antiserum (anti-SmI) to detect protein targets exposed by miltefosine on the surface of treated worms. This serum was developed in a rabbit, a relatively non-permissive host to *S*. *mansoni* infection compared to the more permissive human or murine hosts [71,72]. Antibodies in the rabbit immune antiserum were previously reported to enhance PZQ activity in mice [44] and to partially protect the animals against a challenge *S*. *mansoni* infection when injected into them during the first week of infection [73]. Hence, the anti-SmI rabbit serum may be useful for detecting potential antigenic targets of antibodies that enhance worm mortality caused by the drug treatment or that protect against a new infection. These antigens may be potential candidates for development of a vaccine against *S*. *mansoni* infection. A host treated with miltefosine and that has developed specific antibodies to exposed adult worm tegumental proteins may be resistant to subsequent infection through targeting antigenic epitopes on young migrating schistosomula, leading to destruction or disablement of larval migration in the skin or lungs. This concept has been proposed for praziquantel following observations from field studies indicating that protective immunity against re-infection with schistosomiasis can be developed by cumulative exposure to antigens released from or that become exposed at the surface of worms following treatment with PZQ (post-treatment development of resistance) or of worms naturally-dying in untreated populations (age-dependent immunity) [35,74,75]. On the other hand, surface antigens would not be detectable on intact adult schistosome worms of a new infection in the absence of the drug; hence such mechanism of protection by rabbit antibodies is unlikely to occur at the adult worm stage.

In accordance with our inverted microscopy observations, strong immunofluorescence of the worm surface following treatment with miltefosine was recognised using the anti-SmI antiserum, while no fluorescent staining was detected on untreated worms using the same antiserum or on drug-treated worms incubated with a serum from naive rabbits. The staining was found to increase with higher concentrations of miltefosine and the fluorescence intensity was more than that observed on the PZQ-treated worms; for the latter, the brightest staining was induced by 6 μg/ml. Although schistosome adult worm proteins in the subtegument and deeper tissue might become exposed as a result of drug treatment, the epitopes that appear to be recognized utilising rabbit anti-schistosome antibodies are likely those on or very near the parasite surface as demonstrated by the surface labeling observed in the fluorescent microscopy studies. Miltefosine doses/durations of treatment found to produce adequate unmasking, as indicated by fluorescence staining intensity, were 40 ug/ml for 48 hours and 20ug/ml for 72 hours. These concentrations could be potentially achieved *in vivo* based on previous data on clinical pharmacokinetics of miltefosine against *Leishmania* infections [76], yet smaller doses for longer periods not tested in this work might achieve similar effects.

Rabbit antibodies eluted from miltefosine-treated worms recognised two dominant proteins at ~33 and 23, as did the antibodies recovered from PZQ-treated parasites, yet the reactivities of the latter were of relatively lower intensities. Two additional bands at ~37 and ~120–130 kDa were detectable by the anti-SmI antibodies detached from miltefosine-treated worms, but hardly seen with the antibodies from PZQ-treated worms. Miltefosine thus seems to expose more antigens/epitopes when compared to PZQ.

The immunodominant reactivity at ~33 kDa exposed by treatment with miltefosine was analysed by MS/MS and was found to have peptide sequences that gave 77% sequence coverage of *S*. *mansoni* fructose bisphosphate aldolase. SmFBPA belongs to the class I FBPA family with a predicted molecular size of 39.6 kDa (Uniprot Smp_042160). The enzyme has 65–66% similar amino-acid sequence to human class I C and A isoenzymes [77]. Class I aldolases are characterised by having a (β/α) 8-barrel domain [78], a structure that was reported to be difficult to be completely disrupted by detergents and even high temperature [79]. These properties have been claimed to result in anomalously fast protein migration in SDS-PAGE gels [80]. De Montigny and colleagues [81] detected an aldolase subunit from the eubacteria *Thermus aquaticus* with a mass of 34 kDa on reducing SDS-PAGE, a size lower than the theoretical 41 kDa (homotetramer mass estimated to be 165 kDa). The other possibility is that the protein detected here at ~33 kDa might correspond to a lower molecular-sized SmFBPA isoform. Class I aldolases in eukaryotes have been reported to differ in molecular mass [82], with aldolase I B subunits (~70% homologous to both aldolases I A and C in amino-acid sequence) having a smaller molecular size of 36 kDa [83], a size near to the SmFBPA-band detected in the present work. Two different isoforms of *Schistosoma haematobium* adult worm FBPA were recently characterised and reported to have disparate immunogenic properties [84]. The situation in *S*. *haematobium* may be similar in *S*. *mansoni*; i.e., there may be two different SmFBPA isoforms possessing different immunogenic properties. Further investigations to characterise the relationship between SmFBPA p33 and the previously described SmFBPA p42 [77] are needed. SmFBPA is a tegument protein expressed in different schistosome life cycle stages and plays a central role in glycolysis and generation of the energy needed by the parasite for its survival [77,85]. The enzyme was shown to be recognised by antibodies specific to the lung stage schistosomulum antigens released in culture medium and antibodies from rabbits vaccinated with irradiated cercariae [86,87]. Recombinant- and DNA-vaccines based on SmFBPA induced 41.7–57% protection against *S*. *mansoni* challenge infections and significant reductions in the number of hepatic granulomas in mice [84,87].

IgG antibodies in the anti-SmWH antiserum eluted from PZQ-treated worms showed a different antigenic pattern when compared to all other elutions, in so far as the SmFBPA-containing band at ~33 kDa was not evidenced in the blots, although treatment with PZQ might be predicted to expose the same antigenic epitopes as those recognized by eluted anti-SmI antibodies. These findings may imply that miltefosine acts to unmask epitopes on SmFBPA protein that are different from those exposed by PZQ. The epitopes unmasked by the first were bound by specific IgG antibodies in the anti-SmWH serum following miltefosine treatment. The anti-SmWH antiserum, unlike the anti-SmI, failed previously to enhance the efficiency of PZQ in mice [44]. Since the anti-SmWH antibodies eluted off PZQ-treated worms did not recognise the SmFBPA pr33, our present findings suggest this antigen may have an important role in mediating the PZQ/antibody synergistic effect.

Our MS/MS analysis of the 23 kDa band showed the presence of peptide sequences for the *S*. *mansoni* tegument protein Sm22.6, a cryptic allergen like surface membrane protein (TAL1) with unknown function, although it was thought to inhibit human thrombin [88]. Sm22.6 is a major target of specific IgE antibody in infected and PZQ-treated individuals [89,90]. This antibody response was implicated in resistance to re-infection [89,91,92]. The protein may have an important role in entry into the host vasculature since it becomes critically up-regulated in schistosomula [93]. Recombinant Sm22.6 was shown to induce specific anti-Sm22.6 IgG antibodies in mice and to partly protect the animals against infection [94].

The high molecular weight protein band detected at ~120–130 kDa had a molecular mass similar to that of the dimeric form of *S*. *mansoni* alkaline phosphatase (SmAP) of ~130 kDa [61]. To investigate whether SmAP was exposed by miltefosine treatment, we used a rabbit anti-SmAP-specific antiserum to probe the surface of *S*. *mansoni* worms that had been treated with the drug. Since anti-SmAP antibody was available, providing an opportunity to specifically detect SmAP without the need for mass spectrometry, the antibody was used to identify SmAP. Immunofluorescence analysis confirmed the protein exposure by inducing intense, bright staining of the surface of miltefosine-treated worms compared to the untreated controls. The differences in labeling between untreated control worms treated with the anti-SmAP and the polyspecific anti-infection or anti-worm homogenate sera could be related to different anti-SmAP antibody concentrations and/or epitope specificities between the last two sera and the anti-SmAP antiserum.

Anti-SmAP antibodies eluted from these worms reacted with a protein band of molecular mass similar to that detected by the anti-SmI and anti-SmWH antibody elutions from miltefosine-treated worms. SmAP is a marker for the schistosome adult worm tegument, where its exposure was found to be enhanced by PZQ [95–99], but also present in internal tissues of the parasite [98]. The enzyme was shown to be involved in host invasion, nutrient uptake and immune evasion of host immune responses by generating anti-inflammatory and immunosuppressant molecules such as adenosine [98,100].

MS/MS analysis of the ~37 kDa protein, the fourth band detected following miltefosine treatment, identified the protein as *S*. *mansoni* malate dehydrogenase, SmMDH. SmMDH is an important enzyme involved in anaerobic carbohydrate metabolism through catalysing reduction of oxaloacetate to malate, the main pathway of energy production for the *Schistosoma* parasite [101]. The enzyme is expressed in schistosome adult worms, cercariae and eggs stages [102].

Another three proteins: a tubercle glycoprotein Sm200 [27], an unidentified 27 kDa protein with an esterase activity [44] and a 43 kDa actin [103] were previously reported to be exposed on *S*. *mansoni* adult worms by PZQ. In the present work, reactive protein bands at similar molecular sizes to the above, which were not selected for MS/MS analysis due to their low intensity relative to the 4 studied bands, were also detected in our western blots by antibodies eluted from miltefosine-treated worms. These reactivities were not detected by rabbit antibodies recovered from PZQ-treated parasites, possibly due to different epitopes being exposed by the two drugs.

The presence/accessibility of the drug-exposed epitopes on the surface of 3-hour schistosomula was assessed by an indirect IF assay. Schistosomula were heavily agglutinated following incubation with the rabbit anti-SmI antibodies recovered from worms exposed to PZQ and miltefosine. Schistosomulum-agglutination was most extensive with anti-SmWH antibodies eluted from miltefosine-treated parasites. These results indicate that the epitopes exposed by the drugs on adult worms are also expressed on early schistosomula. This agglutination is supposed to induce parasite immobilization preventing its migration and leading to parasite killing *in vivo* by antibody-dependent cell-mediated cytotoxicity (ADCC) [104–107]. The key feature of radiation-attenuated cercarial vaccines (RA-vaccine) may be the parasite’s slow migration and its subsequent attack by the host immune system in the lung [108]. No agglutination or fluorescence was observed with sera from naive rabbits or animals immunized with adjuvant alone or from the anti-SmWH antibodies recovered from PZQ-treated worms. The latter non-agglutinating antibodies showed reactivities against Sm22.6 and SmAP but not against the SmFBPA pr33 in western blots. The low levels of expression of the first two proteins on 3-hour schistosomula could be an explanation [102,109].

In conclusion, our results suggest that the *S*. *mansoni* proteins FBPA, Sm22.6, alkaline phosphatase, malate dehydrogenase are exposed by miltefosine and PZQ treatment on the surface of adult worms, the degree of exposure of the last two being greater with miltefosine. Future experiments may seek to investigate the combined effect of both drugs on exposure of schistosome worm surface proteins. These antigens could be possibly implicated in a proposed miltefosine-mediated immune-dependent action *in vivo* as specific antibody response against miltefosine-exposed antigens could enhance the drug activity. Results of experiments with sera from *S*. *mansoni* infected humans to investigate whether anti-schistosome antibodies would react similarly to the rabbit antibodies would be instructive. Miltefosine doses and durations of treatment found to produce adequate unmasking of tegumental proteins, as indicated by relative intensity of fluorescence staining in this study, were 40 ug/ml for 48 hours and 20ug/ml for 72 hours. These concentrations could potentially be achieved *in vivo* based on previous data on clinical pharmacokinetics of miltefosine against *Leishmania* infections [76], though exposure to smaller doses for longer periods (not tested in the present work) may achieve *in vivo* the effects observed in our *in vitro* study. Our data predict that SmFBPA pr33 may have a dominant role in drug-mediated immune-dependent effect and could be a principal antigen on the surface of 3-hour shistosomula and thus a target for immunological intervention. Further experiments are ongoing to evaluate the effect of antibody-miltefosine interaction *in vivo* and the individual roles of the recognised antigens in miltefosine-mediated worm destruction.

## Supporting information

S1 Table MASCOT search output of NCBInr with the tandem MS data from the purified ~33 kDa gel band. (DOCX)

S2 Table MASCOT search output of NCBInr with the tandem MS data from the purified 23 kDa gel band. (DOCX)

S3 Table MASCOT search output of NCBInr with the tandem MS data from the purified ~37 kDa gel band. (DOCX)

S1 Fig Tegumental changes in PFA-fixed *S*. *mansoni* adult male worms following *in vitro* praziquantel and miltefosine treatments. (PPTX)

S2 Fig Uncropped blot images for Fig 3(ii). (PPTX)

S3 Fig Uncropped blot image for Fig 4(ii), lane 4. (PPTX)

S4 Fig Determining the molecular weight of SmAP with 8% SDS-PAGE gel. (PPTX)

S5 Fig Uncropped gel and blot images for Fig 5(ii), 37 kDa band. (PPTX)

S6 Fig Uncropped gel and blot images for Fig 5(ii), 23 kDa band. (PPTX)

S7 Fig Uncropped gel and blot images for Fig 5(ii), 33 kDa band. (PPTX)
