# Supplementary material for: Treatment of Schistosoma mansoni with miltefosine in vitro enhances serological recognition of defined worm surface antigens
Source: PLoS Negl Trop Dis. 2017 Aug 25;11(8):e0005853. doi: 10.1371/journal.pntd.0005853 (PMC5589257; doi:10.1371/journal.pntd.0005853)
Supplement: S1 Table — (DOCX) [file pntd.0005853.s001.docx]

**S1 Table. MASCOT search output of NCBInr with the tandem MS data from the purified ~33 kDa gel band.**

| **gi:** 1703248 **Fructose-bisphosphate aldolase [*Schistosoma mansoni*]**  **Mass:** 39963 da  **Score:** 1085 **Matches:** 29 (29) **Sequences:** 23 (23) **emPAI**^a^**:** 18.39  **Protein sequence coverage**: 77% | | | | | | | | |
| --- | --- | --- | --- | --- | --- | --- | --- | --- |
| **Peptide match** | | | **Score** | **Expect** | | **Rank** | | **Unique** |
| K.TLPTLiAER | | | 62 | 0.015 | | 1 | | U |
| R.IAQAICAPGK.G | | | 65 | 0.014 | | 1 | | U |
| R.ALQASVLATWK.G | | | 97 | 9.6e-06 | | 1 | | U |
| K.FEGNMGTTLGDK.S | | | 87 | 1.2e-05 | | 1 | | U |
| R.VTEQVLAFVYK.A | | | 87 | 5.8e-06 | | 1 | | U |
| K.GILAADESTATMGK.R | | | 95 | 1.3e-05 | | 1 | | U |
| K.AYTPQENALATVR.A | | | 87 | 3.5e-06 | | 1 | | U |
| K.KENVHAAQEELLK.L | | | 64 | 6.4e-05 | | 1 | | U |
| K.SDDGKTLPTLLAER.N | | | 97 | 8.5e-06 | | 1 | | U |
| K.GILAADESTATMGKR.L | | | 91 | 4.1e-05 | | 1 | | U |
| K.GILAADESTATMGKR.L | | | 96 | 1.1e-05 | | 1 | | U |
| R.LQQIGVENNEENR.R | | | 60 | 0.00016 | | 1 | | U |
| K.KAYTPQENALATVR.A | | | 76 | 0.00092 | | 1 | | U |
| R.LQQIGVENNEENRR.L | | | 62 | 0.0003 | | 1 | | U |
| R.FQPYLTEAQENDLR.R | | | 96 | 1.2e-07 | | 1 | | U |
| R.FQPYLTEAQENDLRR.I | | | 47 | 0.027 | | 1 | | U |
| ANGAAAVGKFEGNMGTTLGDK | | | 26 | 0.047 | | 1 | | U |
| K.LAENISGVILFEETLHQK.S | | | 90 | 2.1e-06 | | 1 | | U |
| ISSHTPSYLAMLENANVLAR | | | 94 | 1.6e-05 | | 1 | | U |
| K.ISSHTPSYLAMLENANVLAR.Y | | | 95 | 1.3e-05 | | 1 | | U |
| GVVPLAGTDNETTTQGLDDLASR | | | 150 | 3.2e-11 | | 1 | | U |
| TVPPAVPGITFLSGGQSELDATK | | | 84 | 6.9e-07 | | 1 | | U |
| ALADHHVYLEGTLLKPNMVTAGQACK | | | 86 | 4.1e-05 | | 1 | | U |
| ALADHHVYLEGTLLKPNMVTAGQACK | | | 33 | 0.0082 | | 1 | | U |
| VDKGVVPLAGTDNETTTQGLDDLASR | | | 160 | 3e-12 | | 1 | | U |
| ALADHHVYLEGTLLKPNMVTAGQACK | | | 83 | 3.3e-06 | | 1 | | U |
| YASICQQNGLVPIVEPEVLPDGDHDLLTAQR | | | 83 | 4e-08 | | 1 | | U |
| **Percentage sequence coverage:** 77%. Matched peptides are underlined. | | | | | | | | |
| 1 MSRFQPYLTE | AQENDLRRIA | QAICAPGKGI | | | LAADESTATM | | GKRLQQIGVE | |
| 51 NNEENRRLYR | QLLFSADHKL | AENISGVILF | | | EETLHQKSDD | | GKTLPTLLAE | |
| 101 RNIIPGIKVD | KGVVPLAGTD | NETTTQGLDD | | | LASRCAEYWR | | LGCRFAKWRC | |
| 151 VLKISSHTPS | YLAMLENANV | LARYASICQQ | | | NGLVPIVEPE | | VLPDGDHDLL | |
| 201 TAQRVTEQVL | AFVYKALADH | HVYLEGTLLK | | | PNMVTAGQAC | | KKAYTPQENA | |
| 251 LATVRALQRT | VPPAVPGITF | LSGGQSELDA | | | TKNLNEINKI | | PGPKPWALTF | |
| 301 SFGRALQASV | LATWKGKKEN | VHAAQEELLK | | | LAKANGAAAV | | GKFEGNMGTT | |
| 351 LGDKSLFVAN | HAY |  | | |  | |  | |

^a^emPAI, the exponentially modified protein abundance index.
