# Supplementary material for: Treatment of Schistosoma mansoni with miltefosine in vitro enhances serological recognition of defined worm surface antigens
Source: PLoS Negl Trop Dis. 2017 Aug 25;11(8):e0005853. doi: 10.1371/journal.pntd.0005853 (PMC5589257; doi:10.1371/journal.pntd.0005853)
Supplement: S2 Table — (DOCX) [file pntd.0005853.s002.docx]

**S 2 Table. MASCOT search output of NCBInr with the tandem MS data from the purified 23 kDa gel band**

| **gi:** 135578 **Tegument antigen Sm22.6 [*Schistosoma mansoni*]**  **Mass:** 22563 da  **Score:** 1262 **Matches:** 53 (32) **Sequences:** 5 (4) **emPAI**^a^**:** 3.13  **Protein sequence coverage**: 31% | | | | | | | | |
| --- | --- | --- | --- | --- | --- | --- | --- | --- |
| **Peptide match** | | **Score** | | **Expect** | | **Rank** | | **Unique** |
| LPPNIEIIAATMSK | | 103 | | 9.3e-008 | | 1 | | U |
| LSQMEEFIR | | 75 | | 6.5e-005 | | 1 | | U |
| AFLEIDADSNEMIDKQELIK | | 68 | | 0.0002 | | 1 | | U |
| VAVLGASGGIGQPLSLLLK | | 73 | | 0.014 | | 1 | |  |
| ISIEEFCR | | 49 | | 0.025 | | 1 | | U |
| SLLDNTYGR | | 43 | | 0.11 | | 1 | | U |
| **Percentage sequence coverage:** 31%. Matched peptides are underlined. | | | | | | | | |
| 1 MATETKLSQM | EEFIRAFLEI | | DADSNEMIDK | | QELIKYCQKY | | RLDMKLIDPW | |
| 51 IARFDTDKDN | KISIEEFCRG | | FGLKVSEIRR | | EKDELKKERD | | GKFPKLPPNI | |
| 101 EIIAATMSKT | KQYEICCQFK | | EYVDNTSRTG | | NDMREVANKM | | KSLLDNTYGR | |
| 151 VWQVVLLTGS | YWMNFSHEPF | | LSIQFKYNNY | | VCLAWRTPSQ | |  | |

^a^emPAI, the exponentially modified protein abundance index.
