# Supplementary material for: Treatment of Schistosoma mansoni with miltefosine in vitro enhances serological recognition of defined worm surface antigens
Source: PLoS Negl Trop Dis. 2017 Aug 25;11(8):e0005853. doi: 10.1371/journal.pntd.0005853 (PMC5589257; doi:10.1371/journal.pntd.0005853)
Supplement: S3 Table — (DOCX) [file pntd.0005853.s003.docx]

**S3 Table. MASCOT search output of NCBInr with the tandem MS data from the purified ~37 kDa gel band.**

| **gi:** 353230847,**Malate dehydrogenase [*Schistosoma mansoni*]**  **Mass:** 36305 da  **Score:** 469 **Matches:** 54 (8) **Sequences:** 8 (4) **emPAI**^a^**:** 0.67  **Protein sequence coverage**: 31% | | | | | | | | |
| --- | --- | --- | --- | --- | --- | --- | --- | --- |
| **Peptide match** | | **Score** | | **Expect** | | **Rank** | | **Unique** |
| IQEAGTEVVEAK | | 84 | | 0.0014 | | 1 | | U |
| LFGVTTLDVVRSNTFIAQAK | | 80 | | 0.0035 | | 1 | |  |
| AGAGSATLSMAYAGVR | | 80 | | 0.003 | | 1 | | U |
| VAVLGASGGIGQPLSLLLK | | 73 | | 0.014 | | 1 | |  |
| LNEYEIELLK | | 56 | | 0.88 | | 1 | | U |
| FAVSLLEAMSGR | | 56 | | 0.87 | | 1 | | U |
| QSPLISQLALYDIAHVK | | 18 | | 4.4e+03 | | 5 | | U |
| **Percentage sequence coverage:** 31%. Matched peptides are underlined. | | | | | | | | |
| 1 MFSRGANVFR | FGISFRSFLT | | SSKHSPKVAV | | LGASGGIGQP | | LSLLLKQSPL | |
| 51 ISQLALYDIA | HVKGVAADLS | | HIETQAHVTA | | HLGPGELAEC | | LTGANVVIIP | |
| 101 AGMPRKPGMT | RDDLFNTNAS | | IVAELIDSCA | | KNCPKAMICI | | ITNPVNSTVP | |
| 151 IAAEILKRHN | VYDPKRLFGV | | TTLDVVRSNT | | FTAQAKDLAV | | RKVSCPVIGG | |
| 201 HSGITILPVI | SQCSPHVSFP | | QDEREKITKR | | IQEAGTEVVE | | AKAGAGSATL | |
| 251 SMAYAGVRFA | VSLLEAMSGR | | AGVVECAFVE | | SDVTECEFFS | | TPLALGAEGV | |
| 301 EKNMGIGKLN | EYEIELLKKL | | IPELKANIKK | | GKEFAAKYTP | | K | |

^a^emPAI, the exponentially modified protein abundance index.
