# Supplementary material for: Treatment of Schistosoma mansoni with miltefosine in vitro enhances serological recognition of defined worm surface antigens
Source: PLoS Negl Trop Dis. 2017 Aug 25;11(8):e0005853. doi: 10.1371/journal.pntd.0005853 (PMC5589257; doi:10.1371/journal.pntd.0005853)
Supplement: S1 Fig — (PPTX) [file pntd.0005853.s004.pptx]

## Slide 1
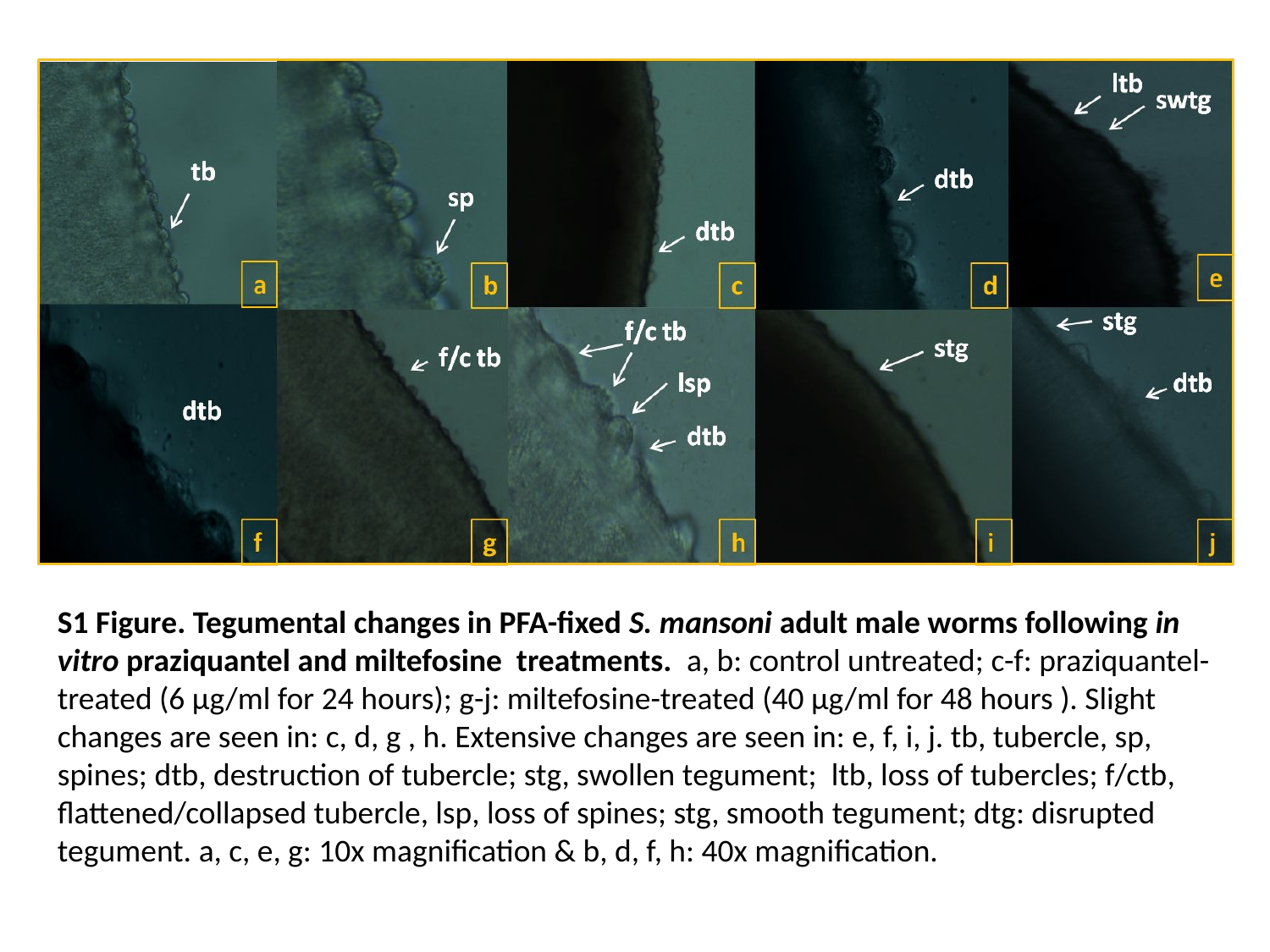

S1 Figure. Tegumental changes in PFA-fixed S. mansoni adult male worms following in vitro praziquantel and miltefosine treatments. a, b: control untreated; c-f: praziquantel-treated (6 µg/ml for 24 hours); g-j: miltefosine-treated (40 µg/ml for 48 hours ). Slight changes are seen in: c, d, g , h. Extensive changes are seen in: e, f, i, j. tb, tubercle, sp, spines; dtb, destruction of tubercle; stg, swollen tegument; ltb, loss of tubercles; f/ctb, flattened/collapsed tubercle, lsp, loss of spines; stg, smooth tegument; dtg: disrupted tegument. a, c, e, g: 10x magnification & b, d, f, h: 40x magnification.
