# Supplementary figures and images for: Treatment of Schistosoma mansoni with miltefosine in vitro enhances serological recognition of defined worm surface antigens
Source: PLoS Negl Trop Dis. 2017 Aug 25;11(8):e0005853. doi: 10.1371/journal.pntd.0005853 (PMC5589257; doi:10.1371/journal.pntd.0005853)

## Slide 1
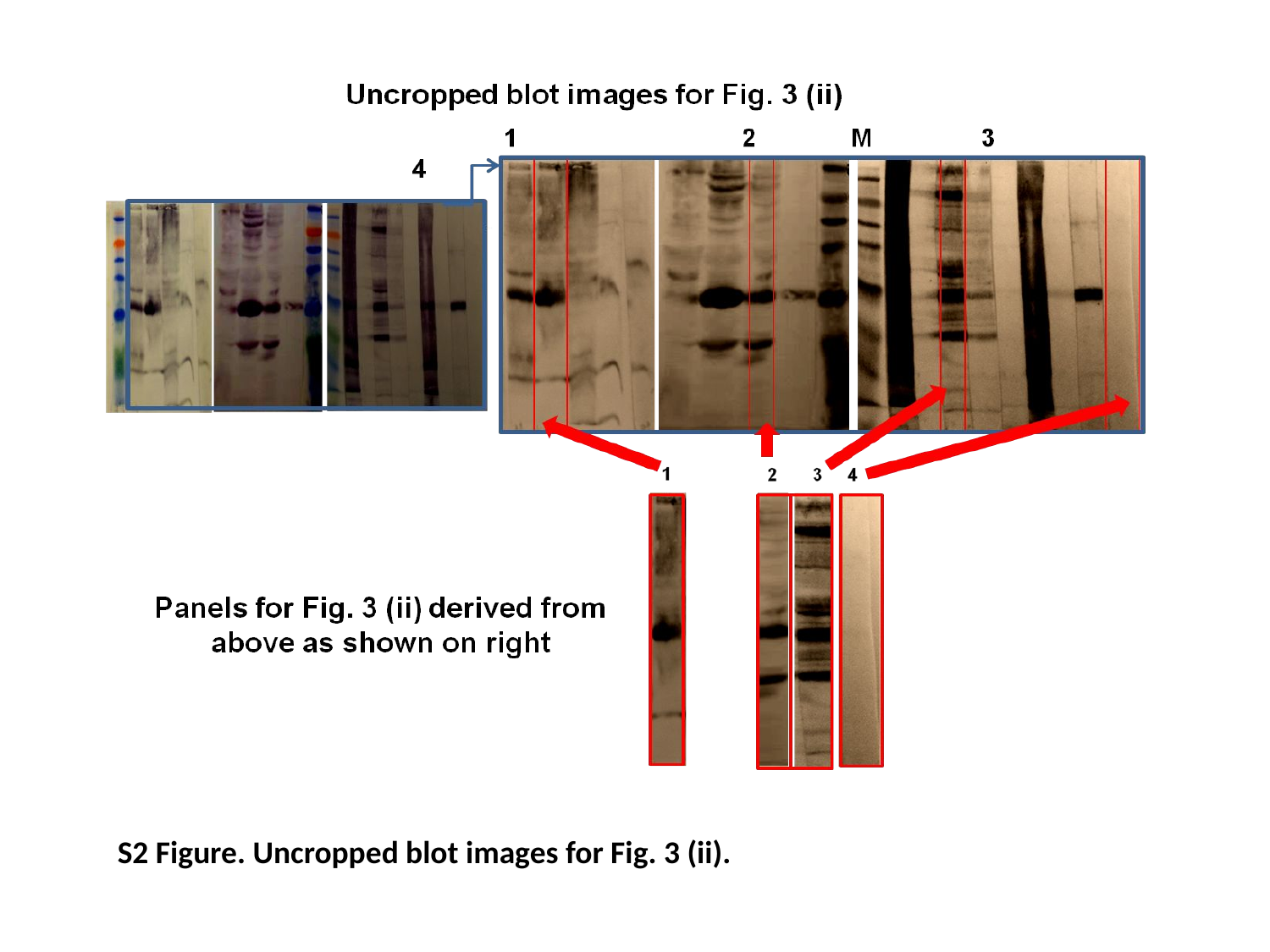

S2 Figure. Uncropped blot images for Fig. 3 (ii).

Supplement: S2 Fig — (PPTX) [file pntd.0005853.s005.pptx]

## Slide 1
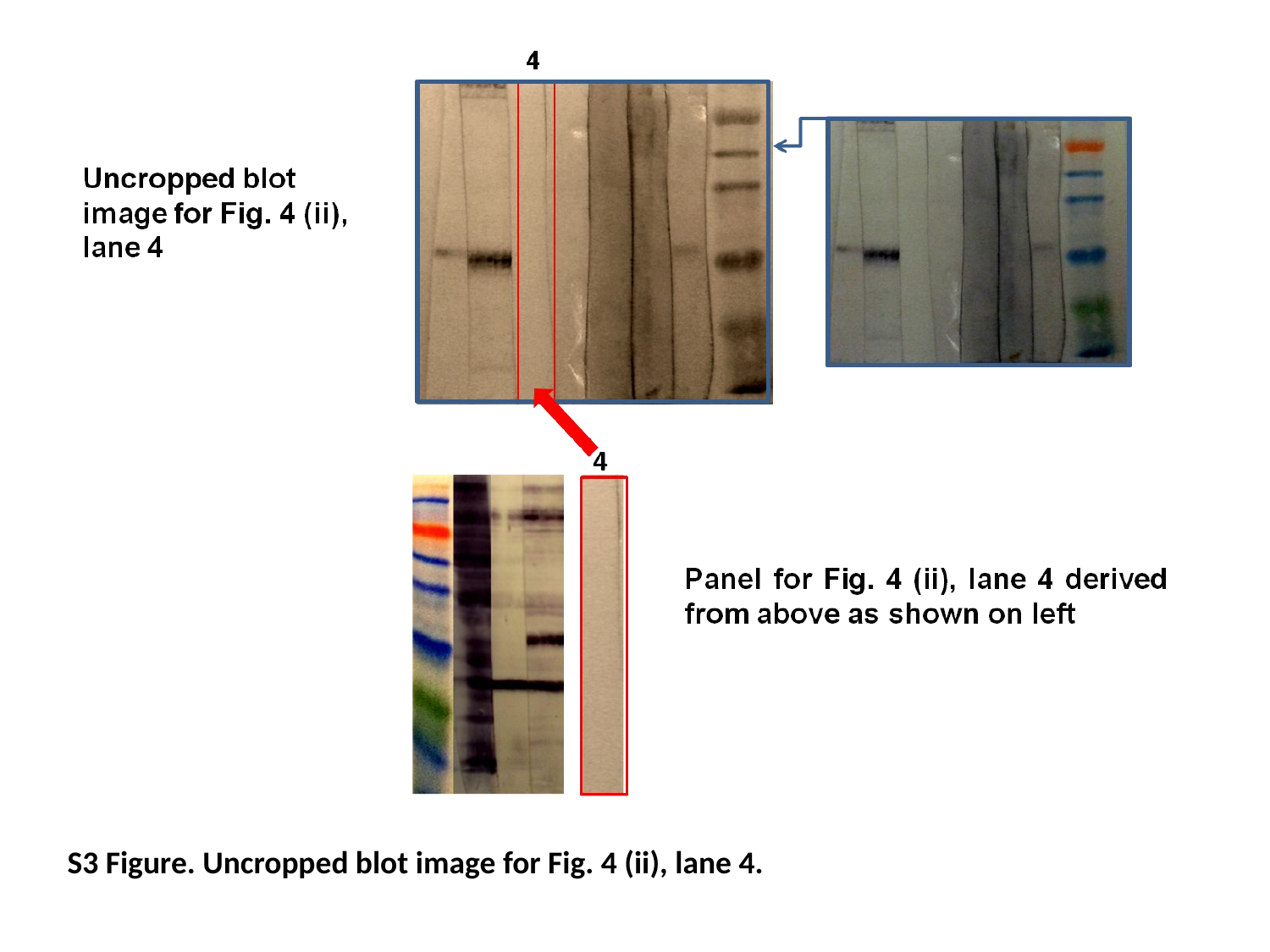

S3 Figure. Uncropped blot image for Fig. 4 (ii), lane 4.

Supplement: S3 Fig — (PPTX) [file pntd.0005853.s006.pptx]

## Slide 1
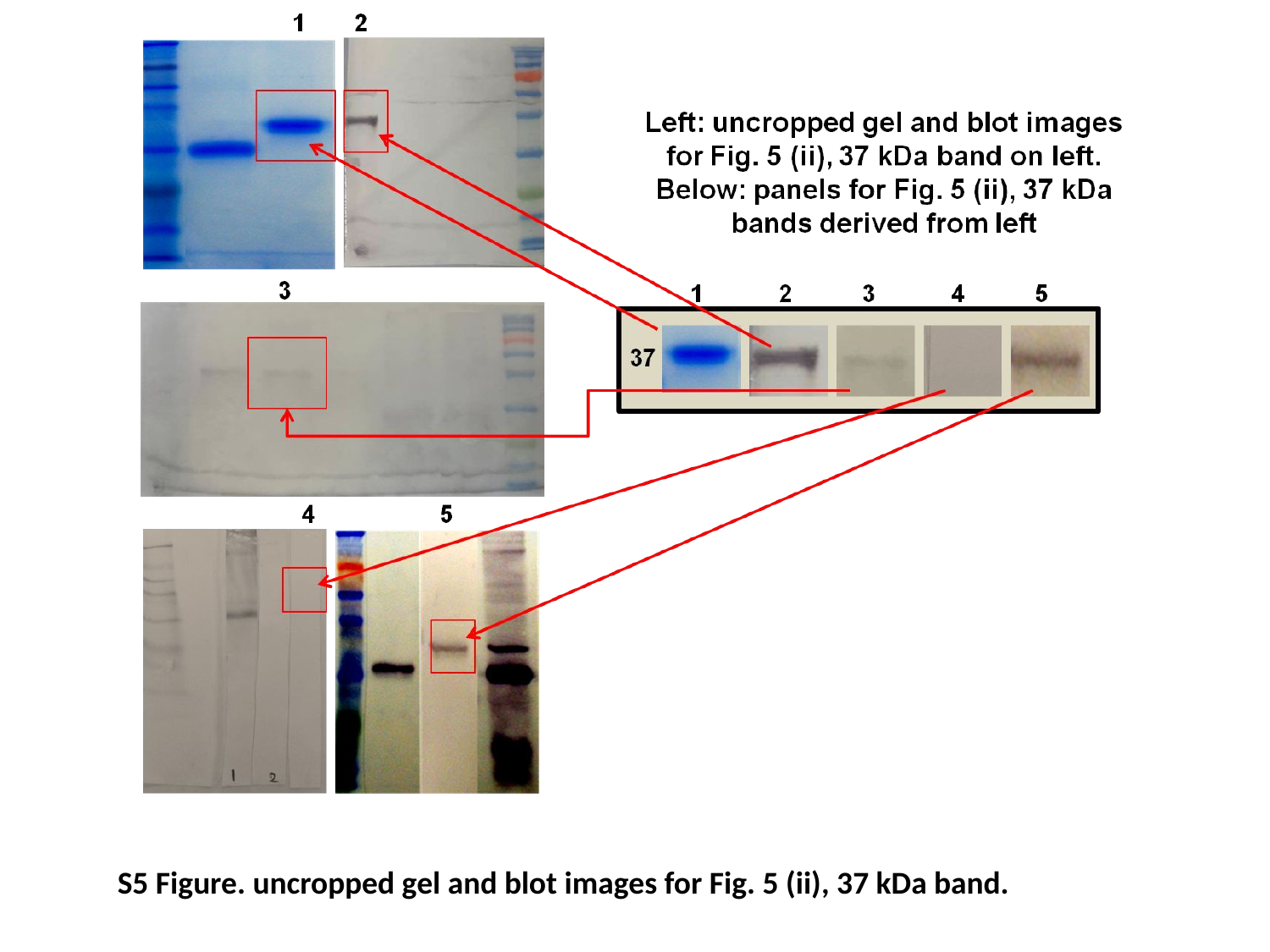

S5 Figure. uncropped gel and blot images for Fig. 5 (ii), 37 kDa band.

Supplement: S5 Fig — (PPTX) [file pntd.0005853.s008.pptx]

## Slide 1
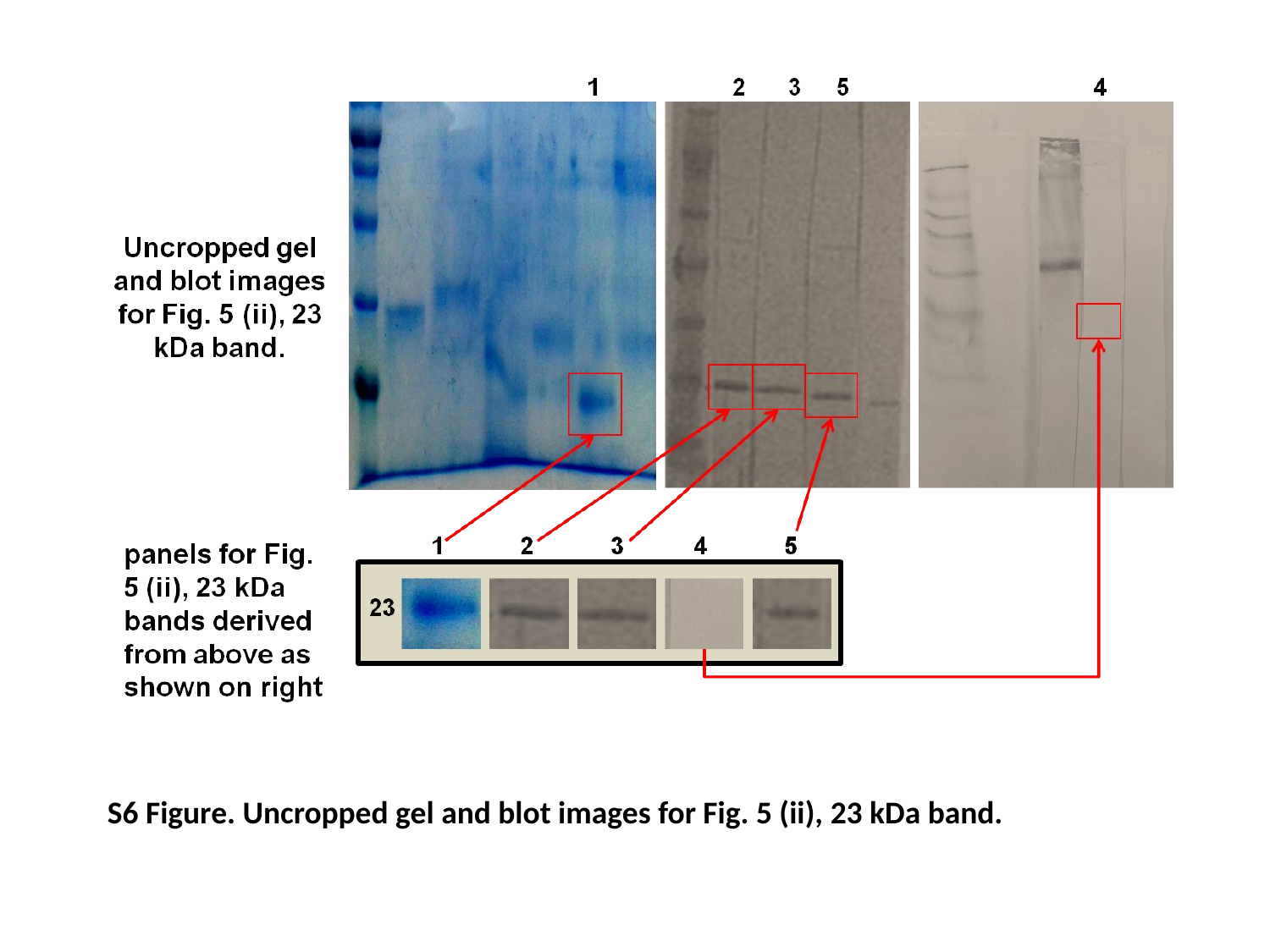

S6 Figure. Uncropped gel and blot images for Fig. 5 (ii), 23 kDa band.

Supplement: S6 Fig — (PPTX) [file pntd.0005853.s009.pptx]

## Slide 1
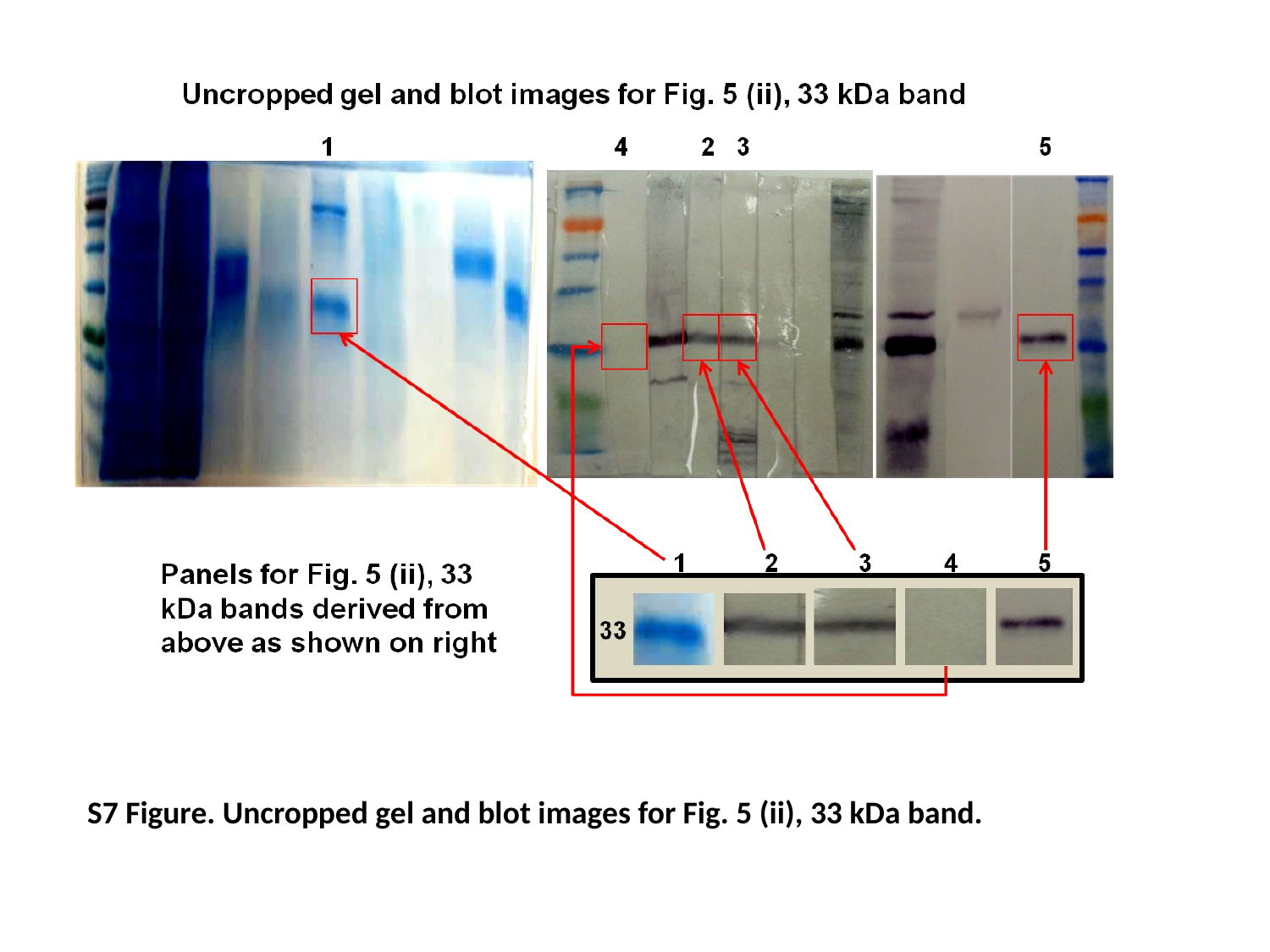

S7 Figure. Uncropped gel and blot images for Fig. 5 (ii), 33 kDa band.

Supplement: S7 Fig — (PPTX) [file pntd.0005853.s010.pptx]
