# Supplementary material for: Treatment of Schistosoma mansoni with miltefosine in vitro enhances serological recognition of defined worm surface antigens
Source: PLoS Negl Trop Dis. 2017 Aug 25;11(8):e0005853. doi: 10.1371/journal.pntd.0005853 (PMC5589257; doi:10.1371/journal.pntd.0005853)
Supplement: S4 Fig — (PPTX) [file pntd.0005853.s007.pptx]

## Slide 1
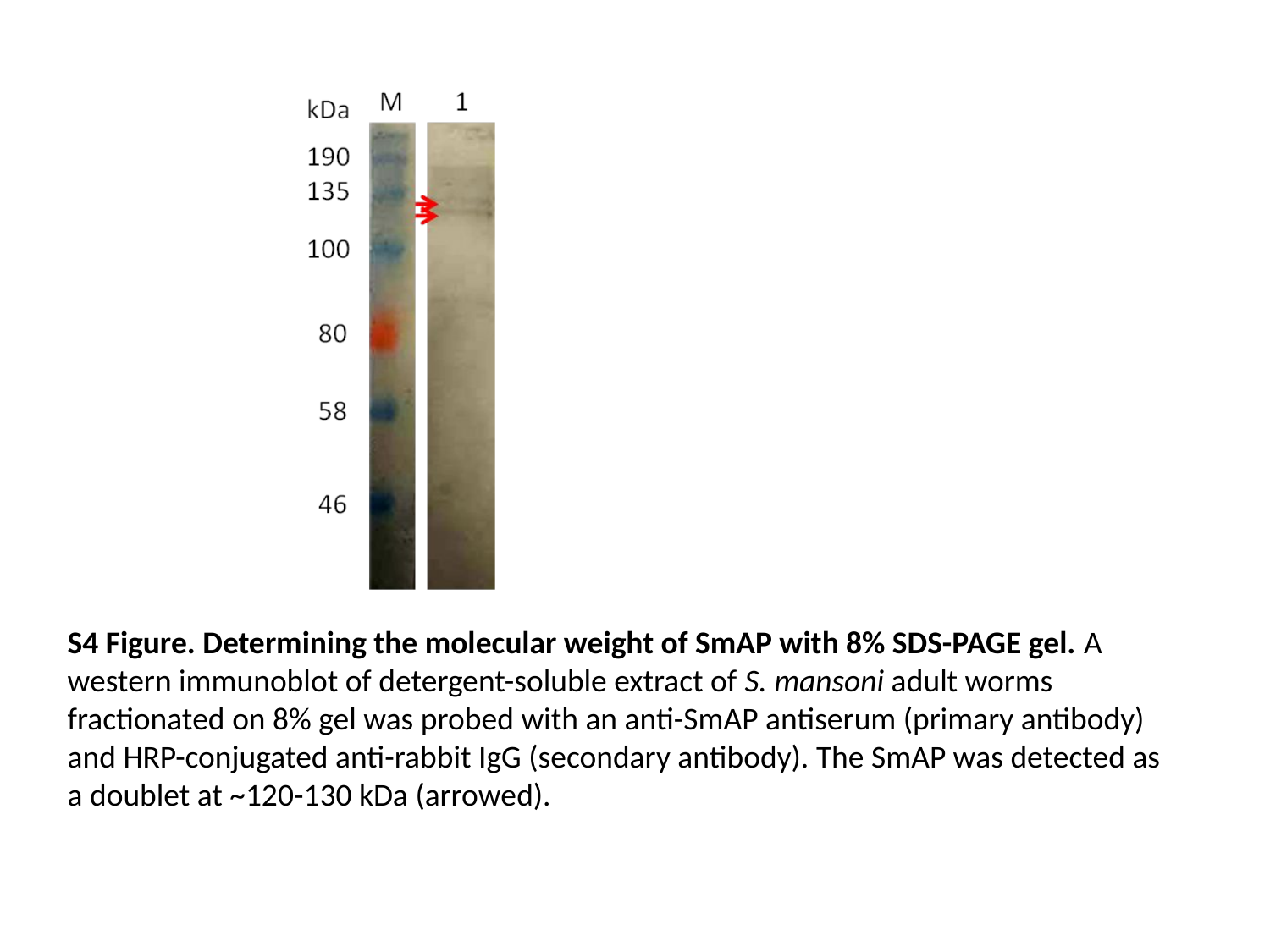

S4 Figure. Determining the molecular weight of SmAP with 8% SDS-PAGE gel. A western immunoblot of detergent-soluble extract of S. mansoni adult worms fractionated on 8% gel was probed with an anti-SmAP antiserum (primary antibody) and HRP-conjugated anti-rabbit IgG (secondary antibody). The SmAP was detected as a doublet at ~120-130 kDa (arrowed).
